# Biophysical signal-driven scaffold design for stem cell-guided osteochondral regeneration

**DOI:** 10.1016/j.bioactmat.2025.12.021

**Published:** 2026-02-16

**Authors:** Yu Gao, Yaling Zhuang, Tongtong Zhu, Hanyang Zhang, Yinan Wang, Fei Chang, Jianxun Ding

**Affiliations:** a Department of Foot and Ankle Surgery, The Second Hospital of Jilin University, 4026 Yatai Street, Changchun, 130041, PR China; b State Key Laboratory of Polymer Science and Technology, Changchun Institute of Applied Chemistry, Chinese Academy of Sciences, 5625 Renmin Street, Changchun, 130022, PR China; c School of Applied Chemistry and Engineering, University of Science and Technology of China, 96 Jinzhai Road, Hefei, 230026, PR China; d Department of Biobank, Division of Clinical Research, The First Hospital of Jilin University, 1 Xinmin Street, Changchun, 130021, PR China; e Department of Traumatic Orthopedics, China-Japan Union Hospital of Jilin University, 126 Xiantai Street, Changchun, 130033, PR China

**Keywords:** Tissue engineering, Scaffold, Biophysical signal, Differentiation of mesenchymal stem cell, Osteochondral regeneration

## Abstract

Osteochondral tissue comprises two structurally and functionally distinct regions: An avascular, low-cellularity cartilage layer with poor self-healing capacity, and a vascularized, mineralized subchondral bone. This pronounced heterogeneity complicates the repair of defects that span both regions. Conventional clinical treatments, such as microfracture and autologous chondrocyte implantation, often fail to restore the native biphasic architecture, leading to disorganized fibrocartilage and poor tissue integration. Tissue engineering has emerged as a promising strategy by integrating mesenchymal stem cells (MSCs) with engineered biomaterial scaffolds. However, spatially directing MSCs toward chondrogenic and osteogenic lineages remains challenging. Beyond biochemical cues, biophysical cues play pivotal roles in modulating MSC fate *via* integrin-mediated mechanotransduction, cytoskeletal remodeling, and mechanosignaling pathways, including TRPV4, Piezo1, and YAP/TAZ. When appropriately encoded within scaffolds, these biophysical cues provide sustained, spatially defined guidance to MSCs. This review summarizes recent advances in scaffold design that leverage mechanobiology to construct biomimetic microenvironments, thereby manipulating lineage-specific MSC differentiation and facilitating layered, stratified osteochondral regeneration.

## Highlights

• Stem cell-loaded scaffolds are essential and durable platforms for osteochondral repair.
• Layered osteochondral architecture requires precise guidance of stem cells toward cartilage and bone formation.
• Structural scaffold design encodes biophysical cues that direct orderly stem cell differentiation.
• Biophysical signals provide targeted and stable regulation with fewer side effects than pharmacological approaches.
• A biophysical signal-driven framework is proposed to engineer scaffolds for spatially organized osteochondral regeneration.

## 1 Introduction

Osteochondral tissue is a mechanically and metabolically integrated unit comprising an avascular cartilage layer overlying a vascularized, mineralized subchondral bone. Distinct gradients in vascularity, matrix composition, and stiffness across this interface make full-thickness osteochondral defects difficult to repair. These structural and compositional variations largely account for the inherently poor regenerative capacity of osteochondral defects, where spontaneous repair typically yields disorganized fibrocartilage rather than hyaline cartilage firmly anchored to structurally competent bone [[1], [2], [3]]. Conventional procedures, such as microfracture and autologous chondrocyte implantation (ACI), offer short-term symptom relief but often fail to re-establish the stratified, mechanically heterogeneous osteochondral architecture required for long-term function [[4], [5], [6]].

Tissue engineering offers a promising alternative by integrating mesenchymal stem cells (MSCs) with engineered biomaterial scaffolds [[7], [8], [9]]. Clinically, MSCs can be harvested from bone marrow, synovium, adipose tissue, and other niches. Among these sources, bone marrow-derived mesenchymal stem cells (BM-MSCs) and adipose-derived stromal/stem cells (ADSCs) are the most widely used cell types for osteochondral repair. BM-MSCs remain the most extensively characterized MSC source, whereas ADSCs are particularly attractive because they can be obtained in large numbers with relatively low donor-site morbidity. ADSCs-based strategies for cartilage repair have already been explored in preclinical and early clinical studies, including intra-articular injection and scaffold-based transplantation [[10], [11], [12]]. However, within a single structure scaffold, it remains challenging to deliver spatial and temporal cues that induce chondrogenesis at the cartilage surface while simultaneously promoting osteogenesis and vascularization in the subchondral region [13]. This mismatch between complex osteochondral biology and relatively simple scaffold design is a central bottleneck in current osteochondral repair.

Beyond classical biochemical stimulation, increasing evidence highlights the essential role of biophysical regulation in directing MSCs' fate and shaping the host response. Relevant physical cues include matrix stiffness and viscoelastic relaxation, pore architecture and interconnectivity, material degradability, ligand type and density, and surface micro- and nano-topography. These cues converge on integrin−focal adhesion kinase (FAK) signaling, cytoskeletal remodeling, and mechano-sensitive pathways, such as transient receptor potential vanilloid 4 (TRPV4), Piezo1, and Yes-associated protein and transcriptional co-activator with PDZ-binding motif (YAP/TAZ) [[14], [15], [16]]. From a design perspective, these pathways indicate that an osteochondral scaffold is not simply a passive support, and its mechanical profile and surface structure are continuously "read" by resident MSCs and translated into lineage decisions and paracrine signals.

At the stem cell level, these biophysical inputs influence mesenchymal progenitors through mechano-sensitive hubs. TRPV4 is a calcium ion (Ca^2+^)-permeable channel that responds to matrix viscoelasticity and volumetric strain in three-dimensional (3D) hydrogels. In MSC-loaded matrices, fast stress relaxation, volume expansion, and TRPV4 activation are closely coupled and drive osteogenic differentiation. In contrast, pharmacological inhibition of TRPV4 reduces osteogenesis even when cells remain well spread [17]. In MSC-derived neocartilage, intermittent activation of TRPV4 enhances glycosaminoglycan (GAG) deposition and improves compressive properties, which links appropriately tuned mechanical stimulation to both lineage progression and functional tissue quality [18].

YAP and TAZ form a second central node that translates stiffness, cell shape, and dynamic loading into gene-expression programs in stem cells. The studies in stem cell mechanobiology show that high substrate stiffness and strong actomyosin tension promote nuclear accumulation of YAP/TAZ and favor osteogenic differentiation. In contrast, softer or more dissipative matrices keep YAP/TAZ largely cytoplasmic, biasing cells toward non-osteogenic fates [19,20]. Mechano-sensitive Piezo channels provide a complementary axis. Piezo1 shows robust activation by high-strain or shear-like stimuli in MSCs and has been linked to enhanced osteogenesis and suppression of bone marrow adipogenesis in BM-MSCs, thereby connecting mechanical loading history to long-term bone strength [21]. Together, these studies suggest that scaffold-level parameters, such as stiffness, viscoelastic relaxation, pore structure, and loading regime, are interpreted by MSCs through defined mechanotransduction programs that bias cell fate and paracrine behavior in cartilage-like *versus* bone-like regions.

Guided by this perspective, the present review discusses how intrinsic mechanical cues, interfacial and topographic cues, and exogenous dynamic cues modulate MSC fate and, in parallel, regulate immune adaptation during osteochondral repair. In contrast to earlier reviews that mainly catalogue biomaterial compositions or focus on biochemical growth factor strategies, this review emphasizes a mechanobiological design framework. Three tiers of biophysical regulation—intrinsic mechanical cues, interfacial and topographic cues, and programmable dynamic stimuli—are explicitly mapped onto the osteochondral unit and its resident stem cells. Rather than offering a purely descriptive summary, this review uses these tiers to derive zone-specific design rules, connect them to existing clinical devices, and underline the importance of synchronizing biophysical cue design with rehabilitation protocols. This layered perspective is intended to serve as a practical roadmap for scaffold engineers and clinicians who aim to translate mechanobiology into functional osteochondral regeneration.

**Scheme 1:**
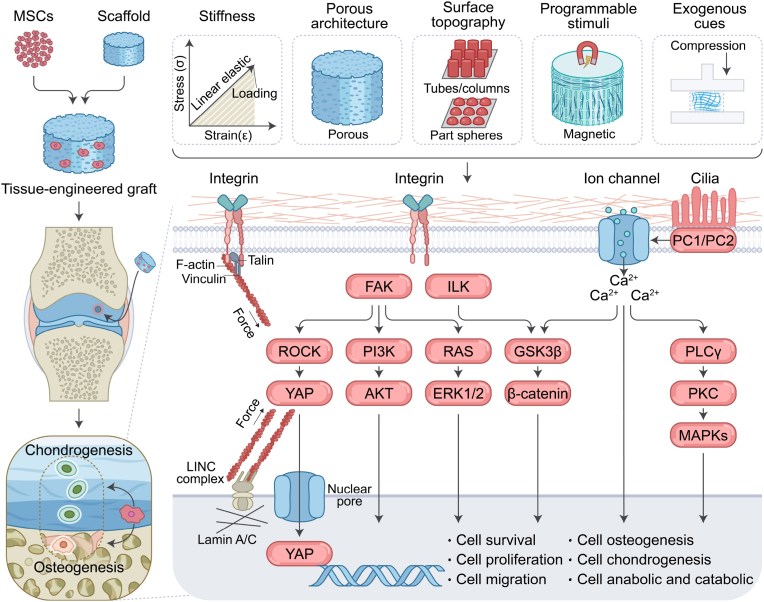
Integrative schematic of biophysical cues and mechanotransduction in osteochondral regeneration.

**Scheme 2:**
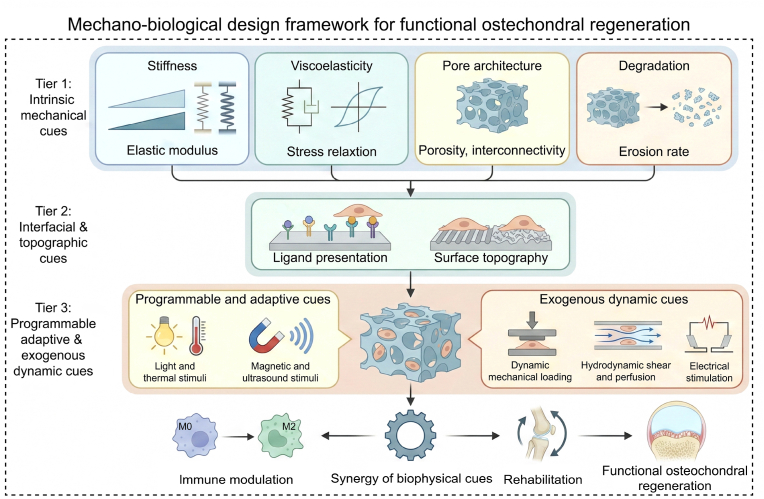
Mechanobiological design framework for functional osteochondral regeneration.

On this basis, the review proposes a mechanobiological design framework for osteochondral scaffolds that seeks to re-establish mechanical and biological continuity between cartilage and subchondral bone by integrating multiscale physical principles. Scheme 1 summarizes the core mechanotransduction pathways, whereas Scheme 2 provides an integrated, stepwise design workflow for osteochondral scaffold development.

## 2 Intrinsic mechanical cues

Intrinsic mechanical cues establish the baseline conditions for osteochondral repair. Stiffness, viscoelasticity, pore structure, and degradation act as key design parameters that influence cellular mechanotransduction and pericellular mass transport. Through coordinated load transfer, these cues protect cartilage during the early phase, promote subsequent bone maturation, and preserve mechanical continuity across the interface.

### 2.1 Stiffness

Stiffness as a central biophysical cue guiding lineage commitment [[22], [23], [24]]. Discher and co-workers first demonstrated that MSC fate is dictated by matrix elasticity: Soft substrates favor soft tissue-like phenotypes, whereas stiffer microenvironments favor bone differentiation. This discovery propelled the development of mechanobiology in regenerative medicine [25]. Within this framework, ranges of stiffness regulate MSC mechano-sensing through integrin-mediated adhesion, nuclear mechanics, and metabolic state, together directing differentiation toward cartilage or bone in the osteochondral unit [26]. On soft matrices, reduced nuclear flattening limits YAP and TAZ nuclear entry and maintains chondrogenic programs, whereas higher stiffness increases lamin tension, drives nuclear accumulation of YAP, and shifts fate toward osteogenesis [27,28]. This increase in stiffness also promotes chondrocyte senescence through an HDAC3-Parkin axis, indicating that scaffolds intended for cartilage repair should avoid premature increases in modulus during the early phase of healing [29]. On the bone side, blood clots provide an elastic and adhesive microenvironment that activates integrin-FAK-Runx2 signaling and accelerates osteogenesis, illustrating that endogenous increases in effective stiffness during early consolidation may be harnessed for repair [15].

Defining stiffness ranges for specific functional zones is a practical strategy for scaffold design. In the cartilage layer, a soft-to-moderate stiffness range of kilopascals supports MSC chondrogenesis [[30], [31], [32]]. Within this stiffness range, adjusting the chondroitin sulfate content while keeping the Young's modulus near several tens of kilopascals further improves cartilage-associated markers in 3D culture [33]. Human stem cells also undergo chondrogenic differentiation in soft hydrogels of a few kilopascals when cell density and matrix composition are favorable, even in the absence of added growth factors [34]. In contrast, the bone layer requires stiffer matrices that not only improve load transfer but also elicit mechanobiological signals that promote osteogenic differentiation of MSCs [[35], [36], [37]].

Recent hydrogel studies provide quantitative stiffness windows directly relevant to osteochondral scaffold design. Using stiffness-gradient hydrogels, Liu et al. showed that MSCs deposited cartilage matrices most effectively in very soft regions with a Young's modulus below 5 kPa, whereas chondrocytes produced more cartilage in stiffer regions above 20 kPa *in vitro* and in a mouse subcutaneous model [38]. This result implies that even within the cartilage compartment, the preferred stiffness niche depends on whether the construct is MSC-based or chondrocyte-based. In 3D bioprinted sodium alginate−gelatin (Na-ALG−Gel) bioinks with moduli of around 50 and 225 kPa, Yang and co-workers observed higher expression of osteogenic markers, such as alkaline phosphatase (ALP) and lipoprotein lipase (LPL), in the stiffer constructs, and inhibition of YAP reduced these responses, highlighting stiffness-dependent mechanotransduction in printed scaffolds [39]. Bu et al. further developed a hyaluronic acid (HA)−Laponite hydrogel that progressively stiffened from roughly 0.8 to 7.4 kPa over 48 h, and this time-evolving stiffness profile enhanced cross-talk between bone marrow stromal cells and endothelial cells and promoted vascularized bone regeneration in a rat cranial defect [40]. In addition, Prouvé et al. reported that poly(acrylamide-*co*-acrylic acid) hydrogels with stiffness around 60 kPa increased osteocyte marker expression, whereas 140 kPa, combined with high stress relaxation, yielded the strongest osteogenic signatures [41]. Together, these data position stiffness as a zonally tunable design parameter. Soft hydrogels in the sub-kilopascal to few kilopascal range favor MSC-driven cartilage formation, whereas stiffer matrices in the tens to hundreds of kilopascals, including dynamically stiffening systems, are more suitable for supporting osteogenic and osteocyte-like differentiation in the subchondral bone region.

**Fig. 1:**
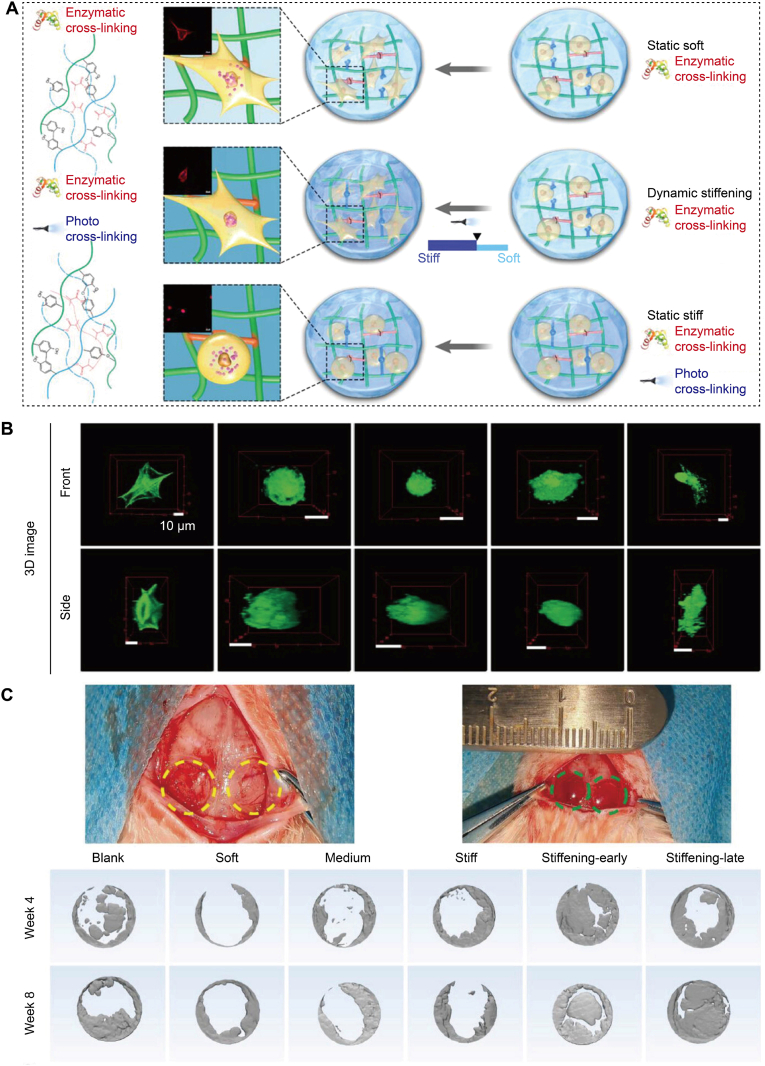
Matrix stiffness dynamics directed MSC behaviors. (A) A dual-functional gelatin network formed a soft state *via* enzymatic cross-linking and could be further photo-cross-linked to yield either dynamic stiffening or a static stiff state, with a static soft control. (B) 3D reconstructions of encapsulated MSCs across groups. Cells spread and enlarged during the soft and early-stiffening phases, whereas stiffer static gels maintained a rounded morphology. Scale bar, 10 μm. (C) Rat calvarial defect implanted with different hydrogels. Micro-CT in weeks 4 and 8 showed more complete and continuous bone fill in dynamically stiffened groups, especially late stiffening, compared with static controls. Reproduced with permission [43]. Copyright 2023, John Wiley & Sons.

Osteochondral healing occurs in sequential phases, making scaffolds with temporally tunable stiffness particularly advantageous [42]. Li et al. developed a dual-functional gelatin network that initially cross-linked enzymatically and subsequently stiffened under light exposure [43], thereby providing an initial soft, permissive phase followed by a stiffer, osteogenic phase (Fig. 1A). In culture, MSCs exhibited extensive spreading and volumetric expansion during the early compliant stage and became increasingly constrained after stiffening, resulting in the enhanced YAP-mediated mechanotransduction (Fig. 1B). The same programmed sequence of stiffening resulted in greater bone volume and better trabecular architecture in a rat calvarial defect, demonstrating that temporally programmed mechanical evolution enhanced osteogenesis *in vivo* (Fig. 1C). Extending this temporal regulation into spatial control, magnetically guided mineral phases were engineered to create continuous or layered gradients of stiffness bridging cartilage and bone, which enhanced interface integration in full-thickness osteochondral defects [44]. Hydrogels with high water content and a gradient in stiffness retain mechanical strength while allowing long-range programming of the modulus, so cells experience a continuous mechanical ramp across depth rather than an abrupt discontinuity in force transmission and maturation [45].

Clinical translation already applies the principle of controlling stiffness. An aragonite-based, cell-free osteochondral implant outperformed microfracture or debridement in randomized cohorts and is now available as an off-the-shelf option [46,47]. This demonstrates that a resorbable yet mechanically competent scaffold supports cartilage repair and subsequent subchondral bone regeneration without the addition of cells or growth factors.

Stiffness acts in concert with other biophysical variables, and its adequate level depends on them. Viscoelasticity and stress relaxation reshape the pericellular load over time and influence the immunomodulatory behaviors of MSCs, so identical bulk moduli may yield different lineage outcomes when dissipation differs [48]. Cyclic mechanical loading activates Yes-associated protein/Rho-associated coiled-coil containing protein kinase (YAP/ROCK) signaling in MSCs, which makes them respond more strongly to a given stiffness level [49]. Strain-controlled organ-on-chip platforms now reproduce strain gradients from cartilage to bone in human cocultures, enabling coordinated control of stiffness, loading schedules, and transport to more closely match exposure histories *in vivo* [50].

The following design guidance summarizes these mechanical principles. Begin with soft to moderate stiffness in the cartilage layer to limit nuclear YAP and preserve chondroprotection, and then increase stiffness locally or globally as cell-secreted matrices accumulate, so that osteogenesis and load transfer across the junction are supported. Use continuous or stepped gradients that mirror the native transition in modulus, and introduce macroporosity to adjust the effective pericellular stiffness without compromising mass transport. Combine stiffness adjustment with viscoelastic relaxation and controlled loading to allow cells to experience realistic sequences of loading, relaxation, and nutrient flow rather than single, isolated conditions.

### 2.2 Viscoelasticity

Viscoelasticity refers to the time-dependent dissipation of stress in a matrix, and this behavior has shifted from a secondary parameter to a primary design variable in osteochondral scaffolds [[51], [52], [53]]. Although early studies emphasized static stiffness, recent studies have shown that cells actively interpret relaxation spectra and loss moduli, reorganizing adhesions, the cytoskeleton, and nuclear mechanics to guide lineage choice and tissue assembly during repair [54]. In collagen (Col)-based matrices that relax on physiologic timescales, MSCs shorten adhesion persistence, remodel actin through ROCK-dependent pathways, and upregulate chondrogenic transcription, establishing rapid stress relaxation as an anabolic cue for the cartilage compartment [55]. Increasing substrate stress relaxation alters stem cell cytoskeletal signaling by slowing actin dynamics and by cyclically activating RhoA [56]. In addition, viscoelastic extracellular matrices broaden epigenetic plasticity through chromatin remodeling, which supports the design of innovative materials for engineering cell fate [57].

Leveraging these viscoelastic principles, recent studies have systematically exploited matrix viscoelasticity as a tunable parameter and the synergistic effects of viscoelasticity with other biophysical cues in osteochondral scaffolds to regulate MSC behaviors and enhance osteochondral regeneration. Liu et al. engineered gelatin methacryloyl (GelMA) hydrogel in which the storage and loss moduli were tuned independently by introducing small dynamic bridges and found that a higher loss modulus, and therefore greater viscous dissipation, promoted BM-MSC spreading, proliferation, osteogenesis, and chondrogenesis while suppressing adipogenesis [58]. In an osteochondral defect model, the viscoelastic GelMA formulation with the highest loss modulus produced the most successful simultaneous repair of cartilage and subchondral bone. Li and colleagues created a family of viscoelastic hydrogels for 3D bioprinting and showed that matching their viscoelasticity to that of bone marrow enhanced BM-MSC proliferation, migration, stemness maintenance, and bone regeneration *in vivo* through an integrin-pFAK-YAP mechanotransduction pathway [59]. In HA-based systems, Kim et al. developed injectable HA−ALG hydrogels with stress relaxation half times from roughly 60 to 2000 s at an elastic modulus near 3 kPa and demonstrated that tuning relaxation behaviors and phosphate content shifted BM-MSC differentiation between hyaline-like cartilage and calcified cartilage, with *in vivo* confirmation of biphasic cartilage formation [60]. Li et al. designed a dynamic HA hydrogel loaded with kartogenin (KGN)-containing microsphere and reported that controlled viscoelasticity, together with sustained release of the small molecule, enhanced focal adhesion formation and chondrogenic gene expression and improved articular cartilage repair in rabbits [61].

**Fig. 2:**
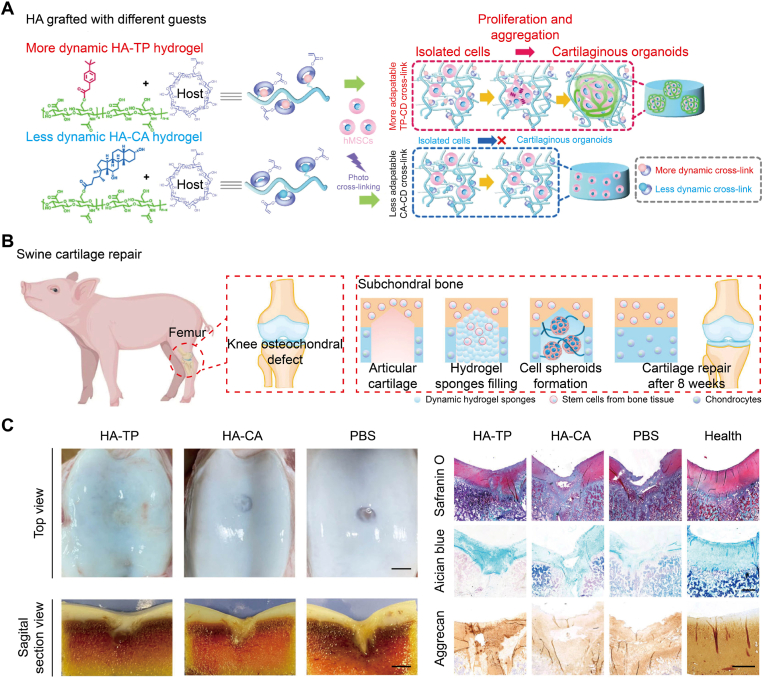
Viscoelastic host−guest dynamics regulated MSC aggregation and cartilage repair [62]. (A) HA networks with different host−guest dynamics. More dynamic HA-TP supported hMSC proliferation and aggregation into cartilaginous organoids, while less dynamic HA-CA kept cells isolated. (B) Porcine knee osteochondral defect model using hydrogel sponges to recruit endogenous cells and induce formation of spheroids, leading to repair after eight weeks. (C) Macroscopic and histological outcomes showed smoother surface and more hyaline-like matrix in HA-TP than HA-CA and PBS, with healthy cartilage as a reference. Stains include Safranin O, Alcian blue, and ACAN. Scale bars, top view 5 mm, sagittal section 2 mm, histology 1 mm, Aggrecan 100 μm.

A recent extensive animal study by Yang et al. engineered an ultra-dynamic host−guest HA hydrogel that programs rapid stress relaxation and staged MSC behaviors [62]. Tuning the guest chemistry defined network dynamics and permitted cell proliferation and aggregation into cartilaginous organoids in the more dynamic formulation (Fig. 2A). In a swine osteochondral defect model, hydrogel sponges filled the lesion and supported *in situ* cell spheroid formation during repair (Fig. 2B). After eight weeks, improved defect filling was observed with stronger GAG staining (Safranin O/Alcian blue) and aggrecan (ACAN) immunoreactivity *versus* controls (Fig. 2C), supporting viscoelasticity as a primary driver of osteochondral healing. On the bone side, Liang and co-workers combined a fast-relaxing viscoelastic hydrogel with cyclic compression to increase MSC osteogenic output and improve healing in a rat femoral defect, demonstrating how controlled dissipation in the subchondral region, when paired with mechanical loading, promotes consolidation [63]. Taken together, these findings show that viscoelasticity and stress relaxation profiles are not secondary details but essential biophysical cues. By tuning loss modulus and relaxation time in different regions of a scaffold, it is possible to bias MSCs toward cartilage-like or bone-like fates and thereby support spatially ordered osteochondral regeneration.

Practical methods now exist to adjust viscoelasticity independently, without altering overall stiffness or ligand density. Light-addressable phototuning accelerates or decelerates stress relaxation by grafting short poly(ethylene glycol) (PEG) tethers into ALG network in space and time, allowing the loss modulus and relaxation half-times to match rehabilitation milestones [64]. Dynamic covalent exchange using cyclic thiosulfinates forms disulfide-based networks that relax *via* thiol−disulfide interchange under physiological conditions, yielding adjustable relaxation spectra while maintaining a nearly constant storage modulus [65]. Supramolecular host−guest HA systems use cucurbit[8]uril-mediated photodimerization to densify or loosen reversible clusters, producing spatially programmable viscoelastic maps [66].

Clinical translation is beginning to reflect these principles, even when product documentation does not explicitly report relaxation spectra. For example, autologous chondrocyte implantation (ACI) products based on hydrogels and associated with extracellular matrices, such as NOVOCART Inject plus, have outperformed microfracture in direct clinical comparisons, and their hydrated, injectable matrices stabilize the defect during the early phase, even though their viscoelastic and relaxation properties are not reported explicitly [67].

For regions on the cartilage side, use fast-relaxing networks with high water content to limit sustained nuclear tension and to support condensation and glycolytic reprogramming. After fibrils align and mechanical loads increase, the relaxation rate increases to match the more organized matrices. For regions on the bone side, maintain a moderately slower relaxation that dissipates peak stresses while allowing traction to persist long enough to engage osteogenic programs. The pair of this relaxation profile with controlled cyclic compression achieves mechanical synergistic effects. Treating viscoelasticity in this way turns dissipation from a passive material property into an active lever for staged, MSC-guided osteochondral regeneration.

Taken together, these findings suggest that viscoelasticity should not be tuned in isolation. The same apparent relaxation half-time may drive stem cells toward distinct fates, depending on the underlying modulus, pore architecture, and degradation profile. Fast-relaxing, relatively soft hydrogels, when anchored within a stiffer, bone-like frame, help maintain a cartilage-mimetic microenvironment. In contrast, slower-relaxing networks with large, interconnected pores prolong cell−matrices traction and tend to support osteogenesis and vascular invasion. In practice, viscoelasticity works in concert with stiffness, porosity, loading frequency and amplitude, and resorption kinetics to shape MSC mechanotransduction. The joint action of these biophysical cues is discussed in more detail in Sections 2.1, 2.3, 2.4, and 6.1.

### 2.3 Porous structure

The structures of pores shape the local mechanical and transport microenvironments that stem cells actually sense by altering stress relaxation, fluid and nutrient exchange, and cell spreading, thereby directing their fate [68,69]. In osteochondral scaffolds, the governing descriptors include pore size, overall porosity, pore shape, and pore orientation, and these features evolve as tissue occupies the scaffold, resetting MSCs' adhesion tension, nuclear morphology, and metabolic state over time [70,71].

Pore size is a primary factor in regulating tissue ingrowth [72,73]. Smaller or more constrained pores help cells attach and remain stable in the early stage, whereas larger and well-interconnected pores on the bone-facing side support perfusion, vascular invasion, and deeper bone formation [74,75]. In honeycomb scaffolds made of carbonate apatite, the extent of pore interconnection and the channel diameter together determine both the depth and direction of bone penetration. Channels of appropriate diameter, arranged along a single axis, guide bone growth deeper in that direction, showing that pore architecture—and not only material composition—governs the effectiveness of bone ingrowth [76]. Creating a fully interconnected honeycomb pore network, rather than leaving isolated cavities, further improves material filling and tissue ingrowth *in vivo* [77].

Pan et al. fabricated bilayered poly(lactic-*co*-glycolic acid) (PLGA) scaffolds with different porosity combinations and showed that a construct with 92% porosity in the cartilage layer and 77% porosity in the bone layer most effectively repaired 4 mm × 5 mm femoral condyle defects in rabbits, yielding the best macroscopic appearance, histological quality, and chondrogenic gene expression among the tested groups [78]. Duan et al. then extended this bilayer PLGA platform and reported that implantation of bilayered porous scaffolds in the same defect model supported stable osteochondral repair up to 24 weeks, with further improvement when the pores were preloaded with allogeneic BM-MSCs [79]. Quantitative analysis of these bilayer PLGA systems, together with subsequent reviews, indicates that pore diameters around 100−200 μm in the cartilage layer and 300−450 μm in the bone layer fall within an optimal window for simultaneously supporting chondrogenesis in the superficial zone and vascularized bone ingrowth in the deeper zone, whereas pores smaller than about 300 μm tend to induce hypoxia and insufficient bone formation [80].

In parallel, Conoscenti et al. created a poly(L-lactic acid) (PLLA) scaffold with a continuous pore size gradient from approximately 70 μm on the cartilage side to more than 200 μm on the bone side and demonstrated in a biphasic microphysiological system that this gradient alone, without exogenous growth factors, guided human mesenchymal stem cells (hMSCs) toward chondrogenic phenotypes in the small-pore region and osteogenic phenotypes in the large-pore region [81]. These data show that the porous scaffold behaves like a physical filter, guiding cells toward the cartilage side and away from the bone side, rather than serving as a passive volume filler.

**Fig. 3:**
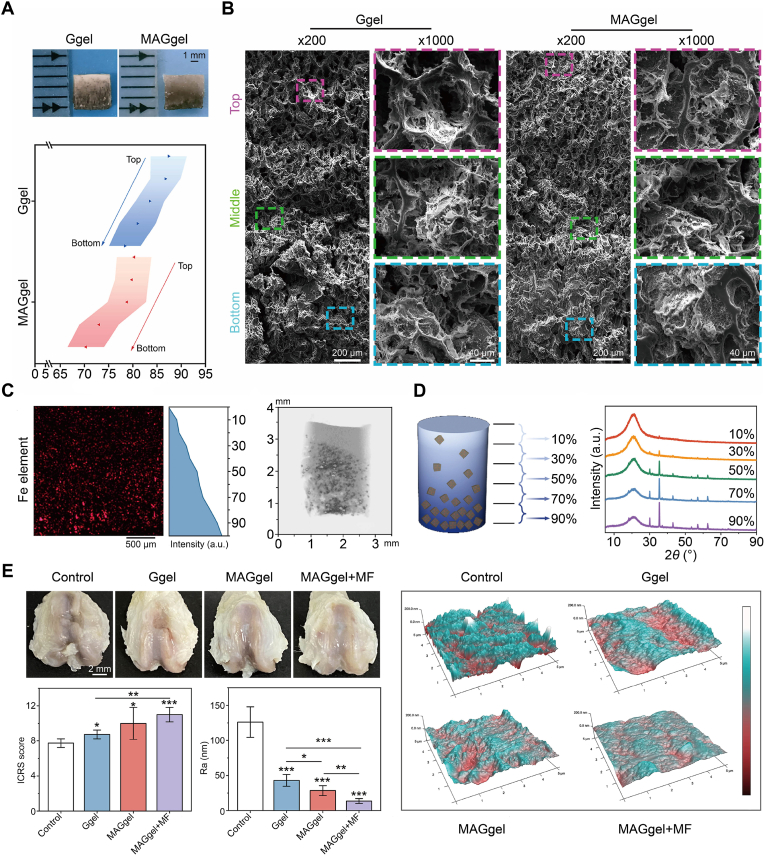
Pore size gradients and mechanical stratification regulated osteochondral repair. (A) Photographs and porosity gradients from top to bottom in Ggel and MAGgel. (B) Scanning electron microscopy (SEM) images showed decreasing pore size from top to bottom, with smaller pores after post-modification in MAGgel. (C) Iron (Fe) distribution mapping and micro-CT confirmed a bottom-enriched particle gradient. (D) Layer-resolved XRD and magnetization demonstrated position-dependent increases consistent with the gradient. (E) Rat knee osteochondral defect repair showed higher ICRS scores and smoother cartilage surfaces for MAGgel and MAGgel with an external magnetic field compared with controls. Scale bars: (A) 1 mm, (B) 200 and 40 μm, (C) 500 μm. All statistical data are represented as mean ± standard deviation (SD; *n* = 4; ∗*P* < 0.05, ∗∗*P* < 0.01, ∗∗∗*P* < 0.001). Reproduced with permission [82]. Copyright 2025, Elsevier.

Building on these quantitative insights, these principles have been translated into continuous architectures that bridge cartilage and bone in a mechanically coherent way. As a representative example, Xu et al. created a magnetic-gradient hydrogel with a continuous depth-wise profile of pores and mechanics that coordinated healing from cartilage to bone. Porosity increased from the bottom to the top (Fig. 3A), a stratified microarchitecture spanned the targeted modulus range (Fig. 3B), magnetic-particle mapping and micro-computed tomography (micro-CT) confirmed a bottom-enriched gradient with spatial responsiveness (Fig. 3C), and position-resolved X-ray diffraction (XRD) verified graded mineralization under external fields (Fig. 3D) [82]. The same construct yielded smoother articular surfaces and higher International Cartilage Repair Society (ICRS) scores than controls (Fig. 3E). The ordered carbonate-apatite honeycomb scaffolds with continuous, open channels supported bone ingrowth, indicating that maintaining a unidirectional, interconnected architecture benefited both regeneration and function [83]. Similarly, studies on triply periodic minimal surface (TPMS)-based bone scaffolds showed that adjusting pore size and spatial distribution—from more closed, pressure-retaining pores to larger, better-connected pores—helped balance mechanical support and tissue ingrowth across regions of a construct [84].

Translating these design rules into fabrication offers several practical routes. Melt-electrowriting and related additive manufacturing provide precise control of pore size and orientation for zone-specific guidance [85,86]. Field-guided casting implements continuous gradients in pore mechanics that may be adjusted after implantation to match rehabilitation demands [82]. Cryogel-based fabrication, by creating highly interconnected macroporous networks without altering the polymer chemistry, improves early cell infiltration and mass transport in scaffolds for cartilage and bone regeneration [87]. Clinically, aragonite-based cell-free osteochondral implants embody this logic. A resorbable interconnected mineral framework, corroborated by randomized mid-term evidence, indicates that pairing porous architecture with appropriate mechanics and degradation provides an off-the-shelf path to repair [88].

In practice, match pore size and interconnectivity to the intended history of pressure, relaxation, and transport in each zone. Use continuous gradients to avoid impedance mismatches at the cartilage−bone interface and report comparable structural and transport metrics, including size distributions, orientation, interconnectivity, and effective diffusivity.

### 2.4 Degradation

Among the biophysical cues encoded in osteochondral scaffolds, degradation is the one that changes most dramatically with time. As chemical bonds are cleaved and the material is resorbed, stiffness, stress-relaxation behavior, and pore architecture all undergo gradual changes. Two constructs that start with the same initial modulus may therefore exhibit significantly different mechanical properties to cells weeks or months later. In 3D hydrogels, for example, progressive network degradation increases stress relaxation and mesh size, allowing cells to spread, migrate, and remodel rather than remaining trapped in an elastic cage [89]. For osteochondral repair, this time axis is particularly critical because cartilage and subchondral bone regenerate on different schedules and bear different loads. A scaffold that degrades homogeneously, therefore risks being "too fast" for one compartment and "too slow" for the other. To manage this, materials are often grouped into fast- and slow-degrading regimes and aligned with the "stiffness brackets" of cartilage and bone along depth [[90], [91], [92]]. In practice, most osteochondral constructs integrate two distinct phases: A cartilage phase and a bone phase. The cartilage phase is hydrogel-like and degrades relatively fast, permitting early cell infiltration and matrix deposition. The bone phase is either mineralized or lattice-like, degrading more slowly to preserve architecture and load sharing during ongoing osteogenesis [93].

The degradation kinetics of 3D networks thus act as a dynamic boundary condition that regulates cell fate. In cartilage-directed PEG hydrogels, tuning the protease-sensitive cross-linker so that cell-mediated degradation tracks the time course of human MSC chondrogenesis permits GAG and type II collagen (Col II) to be deposited throughout the construct. In contrast, non-degradable PEG keeps the matrix confined around cells and suppresses chondrogenic marker expression [94]. When scaffold resorption on the cartilage side lags behind tissue formation, the remaining artificial matrices mechanically and sterically block complete defect filling with cartilage-like tissue. If subchondral zone degradation outpaces bone formation, load-bearing support is lost, and the interface becomes vulnerable. Together, these scenarios outline a simple design rule: Scaffold resorption should be matched as closely as possible to the gradual build-up of new cartilage and bone in bioadaptable osteochondral systems [95].

Layered and gradient scaffolds put this rule into practice. Early in the repair process, a relatively soft, highly permeable region enables stem cells to infiltrate and deposit an initial matrix. Subsequently, *in situ* reinforcement enhances stiffness to meet the requirements of long-term joint loading and integration. This reinforcement often takes place through secondary cross-linking, mineral deposition, or a combination of both [96]. For the subchondral zone, tissue engineering studies converge on the recommendation that biodegradable scaffolds provide sufficient mechanical strength and interconnected porosity to preserve defect geometry while new bone gradually replaces the scaffold at a comparable pace [97]. When a single construct spans cartilage and bone, continuous gradients in degradability and porosity help reduce interfacial mismatch and maintain coherent load transfer during maturation. Despite these conceptual advances, many preclinical reports still track scaffold degradation and remodeling only up to 8−12 weeks and devote limited attention to systemic or joint-level biosafety, leaving the genuine long-term window comparatively under-documented.

**Fig. 4:**
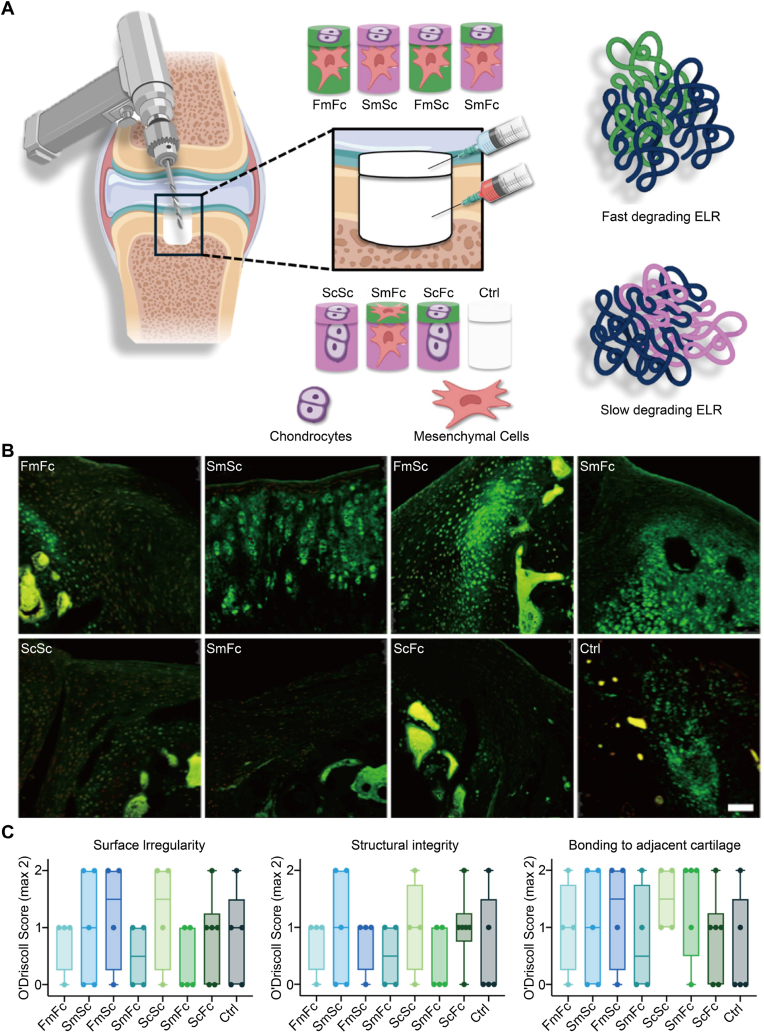
Spatiotemporal degradation cues governed osteochondral regeneration [98]. (A) Surgical implantation and experimental groups, where the two-letter code denotes inner and outer layers, respectively, F or S indicates fast or slow degrading, and m or c indicates MSCs or chondrocytes, and Ctrl denotes untreated defect. (B) Representative fluorescence images of regenerated cartilage across groups with cartilage matrix staining in green. Scale bar, 100 μm. (C) Modified O'Driscoll evaluation showing sub-scores for surface irregularity, structural integrity, and bonding to adjacent cartilage. Box-and-whisker plots show the maximum and minimum values, the interquartile range, and the median (*n* = 4−6).

Recent *in vivo* studies have begun to fill this gap by explicitly programming depth-dependent degradation and following outcomes for six months or longer. Venegas-Bustos et al. created an injectable elastin-like recombinamer hydrogel with a fast-degrading layer on the cartilage side and a slow-degrading layer on the bone side, enabling *in situ* conformal filling of osteochondral defects and scheduling early cartilage preservation followed by subchondral consolidation (Fig. 4A) [98]. After six months *in vivo*, Col II staining shows hyaline-like matrices in the optimized, layered formulations (Fig. 4B). Modified O'Driscoll subscores indicate smoother surfaces, greater structural integrity, and stronger bonding to adjacent cartilage than in controls, linking programmed degradability to *in vivo* benefit (Fig. 4C).

In parallel, Yang et al. combined a photo-cross-linked chondroitin-sulfate hydrogel with a 3D-printed porous pure zinc (Zn) frame to create a bilayer scaffold for full-thickness trochlear defects in Bama mini-pigs [99]. In that study, the Zn layer degraded gradually over 24 weeks as new bone infiltrated it. Magnetic resonance imaging (MRI) and gross inspection revealed near-normal restoration of the articular surface. Importantly, key indicators remained within physiological ranges: Synovial interleukin-1β, synovial tumor necrosis factor-α, serum zinc ion (Zn^2+^) concentrations, and histology of the heart, liver, spleen, lungs, and kidneys. Taken together, these models show that spatially graded degradation may be extended to a six-month window without obvious safety issues. This is achievable as long as corrosion and matrix turnover are closely aligned with tissue formation.

Clinical series with biomimetic, multilayer Col−hydroxyapatite scaffolds (MaioRegen-type constructs) extend these observations into human knees. Prospective cohorts and comparative studies report clinically meaningful improvements in International Knee Documentation Committee (IKDC) and Tegner scores that are maintained for at least 2−3 years after implantation, with relatively low rates of clearly scaffold-related complications [100,101]. Some mid-term series and registry data also suggest that these gains persist for around five years in selected patients. At the same time, second-look arthroscopy and MRI repeatedly show incomplete defect filling or fibrocartilaginous repair in some cases, and failures requiring revision continue to be reported, underscoring that overly slow resorption or interfacial mismatch become clinically relevant. Aragonite-based, cell-free osteochondral implants (Agili-C) offer a complementary perspective. In a pivotal multicenter randomized controlled trial, patients with focal cartilage and osteochondral lesions allocated to Agili-C showed superior lesion filling on MRI and significantly better patient-reported outcomes at 24 months compared with microfracture or debridement, without an excess of serious adverse events (US Food and Drug Administration (FDA) Summary of Safety and Effectiveness Data, P210034). Follow-up reviews and multicenter case series indicate that these clinical benefits can be sustained, but also describe sporadic cases of implant non-integration, subchondral bone cysts, and graft failure, again reminding us that even regulated scaffolds fail when local mechanics, biological remodeling, and degradation become uncoupled [46,102].

From a materials perspective, biosafety considerations extend beyond bulk mechanics to the chemistry and clearance of degradation products. Synthetic polyesters, such as poly(lactic acid) (PLA), poly(glycolic acid) (PGA), and PLGA, are widely used because they ultimately break down into lactic acid and glycolic acid, which enter the tricarboxylic acid cycle or are renally eliminated, and are therefore considered systemically biocompatible [103]. If acidic oligomers accumulate faster than they diffuse away and are buffered, however, they lower local pH, activate macrophages and other immune cells, and impair matrix deposition—effects that have been documented in bone- and cartilage-related settings and are particularly relevant in the confined microenvironments of subchondral bone or a joint cavity [104]. These concerns have motivated the incorporation of basic bioceramics or other buffering components, *e.g*., calcium phosphate (β-TCP), into polyester-based osteochondral scaffolds to temper local acidosis while the polymer phase resorbs.

Biodegradable metals follow a different route: Magnesium (Mg) alloys corrode relatively rapidly and evolve hydrogen gas, which form transient gas pockets and compromise mechanical integrity if corrosion is not controlled [105]. Zn-based metals corrode more slowly and do not generate hydrogen gas in physiological microenvironments, but the therapeutic window for Zn^2+^ is narrower, so alloy composition, corrosion rate, and exposed surface area must be carefully tuned to avoid cytotoxic ion levels while still allowing progressive load transfer to neo-bone [106].

Taken together, these data suggest that degradation should be tuned not only to match the tempo of cartilage and bone regeneration but also to minimize late-stage complications, such as particle-induced synovitis, cyst formation, or subchondral collapse. In practical terms, faster-degrading layers on the cartilage side and slower-degrading, load-bearing layers on the bone side should be aligned with the respective rates of matrix deposition and mineralization, with gradual transitions across depth to preserve mechanical and transport continuity. Long-term validation ought to move beyond simple defect-filling scores and static histology to include quantitative readouts of scaffold volume loss, interface strength, synovial-fluid biomarkers, systemic ion or metabolite levels, and major-organ histology. Treating such long-term safety endpoints as core design criteria—rather than optional extras—will be essential for the next generation of biophysical-signal-driven osteochondral scaffolds.

Across these intrinsic mechanical cues, a few material families play complementary roles in osteochondral scaffold design. Hydrogels, such as GelMA, HA-based networks, and ALG-containing bioinks, are particularly suited for the cartilage side, where high water content, tunable stiffness, and viscoelastic relaxation support chondrogenesis of MSCs and chondrocytes and help to preserve a lubricated articular surface. In contrast, polymer−ceramic composites and bioceramic-rich scaffolds, including Col−hydroxyapatite multilayers, PLGA or PLLA matrices combined with calcium phosphates (CaP), and aragonite-based implants, provide higher stiffness, osteoconductivity, and buffering of acidic degradation products in the subchondral bone region. Emerging degradable metals, such as porous Zn-based constructs, add temporary load-bearing capacity and controlled ion release, while their interconnected architectures permit bone ingrowth and vascularization. Framing stiffness, viscoelasticity, pore architecture, and degradation in terms of these material families helps translate the mechanobiological design rules into practical scaffold choices for osteochondral regeneration.

## 3 Interfacial and topographic cues

Interfacial and surface topographic cues determine how cells interpret and transmit mechanical information during osteochondral repair. Ligand presentation and surface topographic patterns serve as the primary axes for design. Together, they promote integrin clustering and refine cell adhesion. They also modulate nuclear mechanics, steer cell migration, and support matrix alignment.

### 3.1 Ligand presentation

Ligand presentation on biomaterials, especially the display and enrichment of integrin-binding motifs, regulates integrin activation and clustering [[107], [108], [109]]. This conversion turns mechanical cues into biochemical signals that govern cell adhesion, survival, and differentiation [[110], [111], [112]].

Cells detect both the amount of ligand and its organization at the nanoscale. Experiments with titanium dioxide nano-biopatterning showed that specific nanometer-scale ligand spacings promote larger adhesions, greater cell spreading, and higher YAP nuclear localization than sparser or more uniform displays. Membrane-based reconstitution of talin and vinculin further demonstrated that focal-adhesion proteins cluster cooperatively at the membrane to create a platform for force transmission, and nuclear-compression studies linked these mechanically driven adhesion states to YAP dynamics [[113], [114], [115]]. Integrin-ligand specificity works together with nanoscale organization. Col-binding integrins, such as α2β1, are associated with matrix remodeling and with bone-related responses. In contrast, cartilage and joint tissues tend to use α10β1 and α11β1 integrins to bind Col, thereby supporting chondrocyte adhesion and joint homeostasis. Curvature-sensitive adhesion on soft ECM fibers also shows that the physical form of these ligands helps determine which integrin complexes assemble [116,117]. Strain-dependent redox modification of fibronectin fibers alters their mechanochemical behaviors and primes a switch in integrin engagement. This finding shows that ligand chemistry is remodeled by mechanical force and by oxidative cues. The remodeled ECM then feeds into mitochondrial and metabolic regulation, linking these ligand-level changes to broader cellular responses [118,119].

Cartilage-facing regions work best with ligand presentations that permit attachment and orientation but limit excessive spreading. Studies on curvature-controlled adhesions on soft ECM fibers and on fibronectin gradients that guide mesoderm remodeling both show the same pattern. When integrin engagement is modest and spatially organized, tissue structures in these softer zones are easier to maintain [116,120]. On the bone-facing side, incorporating integrin-binding motifs and Col-mimetic peptides into synthetic hydrogels strengthens MSC adhesion and supports osteochondral or bone formation. The hydrogel loaded with MSCs and conjugated with GFOGER promotes osteochondral regeneration, and integrin-dependent mechanotransduction is shown to drive MSC osteogenesis [[121], [122], [123]]. WYRGRL-modified cerium-oxide nanoparticle that targets cartilage tends to accumulate in the cartilage layer, thereby reducing oxidative damage in osteoarthritis *in vivo*. This result shows that sequence-specific targeting helps concentrate therapeutics in the chondral zone [124]. Beyond static cues, photo-responsive hydrogels fabricated by digital light processing allow user-defined, time-sequenced exposure of adhesive and mechanical features. This setup activates, or strengthens, cell−material interactions at chosen times and locations [125].

Taken together, ligand presentation provides the bridge between mechanical signals and cell fate across the osteochondral unit. In practice, ligand density and clustering should be matched to the stiffness and relaxation ranges of each zone. In cartilage layers, α10- or α11-leaning motifs, or motifs that interact with ACAN, together with submicron patterning is used to support condensation and low nuclear strain. In bone layers, Col-mimetic sequences and high-affinity RGD clusters are deployed to extend adhesion lifetime and to increase nuclear tension. The light-responsive or chemically responsive switches are incorporated. This arrangement allows ligand cues to track scaffold degradation and adapt to changing mechanical microenvironments.

### 3.2 Surface topography

Surface topography provides a geometric framework that influences how MSCs attach, organize their cytoskeleton, and sense forces at the interface. At the nanoscale, features typically range from tens to a few hundred nanometers and are comparable to integrin clusters and focal adhesions. At the microscale, ridges, pits, pillars, and channels range from micrometers to tens of micrometers and reshape whole-cell morphology and nuclear architecture. Together with matrix stiffness, these multiscale topographical cues steer MSC differentiation and immunomodulatory behaviors, thereby providing a direct handle for osteochondral scaffold design.

Nanoscale topographies tend to affect early adhesion and intracellular tension. Defined nanopatterns that reduce cytoskeletal contractility were shown to maintain MSC multipotency and sustain an immunomodulatory phenotype by shifting metabolism toward oxidative glycolysis [126]. This work demonstrated that nano-engineered surfaces not only biased lineage choice but also preserved the paracrine functions of MSCs relevant to joint inflammation control. For osteogenic regulation, micro- and nanotopography on titanium and its oxides provides a representative model. Titanium dioxide nanotube arrays with diameters of approximately 100 nm increased periostin expression in BM-MSCs and activated focal adhesion kinase and β-catenin signaling *via* integrin αv, thereby promoting osteogenic differentiation and matrix mineralization [127]. These data support the view that nano-roughened or nanotubular features on the bone-facing side of an osteochondral scaffold can be used to raise local integrin engagement, enhance periostin signaling, and bias MSCs toward stable osteogenesis.

Microscale topographies reshape cell and nuclear geometry and influence stem cell fate through chromatin organization. Studies on the topographic orientation of regenerative scaffolds show that aligned *versus* random microstructures alter how cells distribute, elongate, and assemble extracellular matrices, with vertically aligned scaffolds supporting cartilage and subchondral bone regeneration more effectively than randomly oriented constructs [128]. Recent mechanistic studies further demonstrate that microgrooved or fibrous substrates regulate nuclear tension in MSCs and generate site-specific chromatin accessibility. Aligned microtopographies create anisotropic nuclear stress that promotes gene programs linked to tendon- and muscle-like fates, whereas more isotropic microtopographies increase accessibility at osteogenic and chondrogenic loci and prime MSCs toward skeletal phenotypes [129]. In a cartilage-oriented context, defined substrate geometries, including nano-pillars and nano-holes on polycaprolactone films coated with cartilage matrix components, guided human MSC chondrogenesis and produced distinct cartilage phenotypes with favorable Col II and proteoglycan deposition [130]. These findings indicate that micro- and nano-scale patterns do not play interchangeable roles. Nanoscale features primarily tune focal adhesion signaling and intracellular tension, while microscale patterns regulate cell alignment, nuclear shape, and chromatin remodeling. The two levels converge on lineage-specific transcriptional programs that distinguish cartilage-like from bone-like outcomes.

**Fig. 5:**
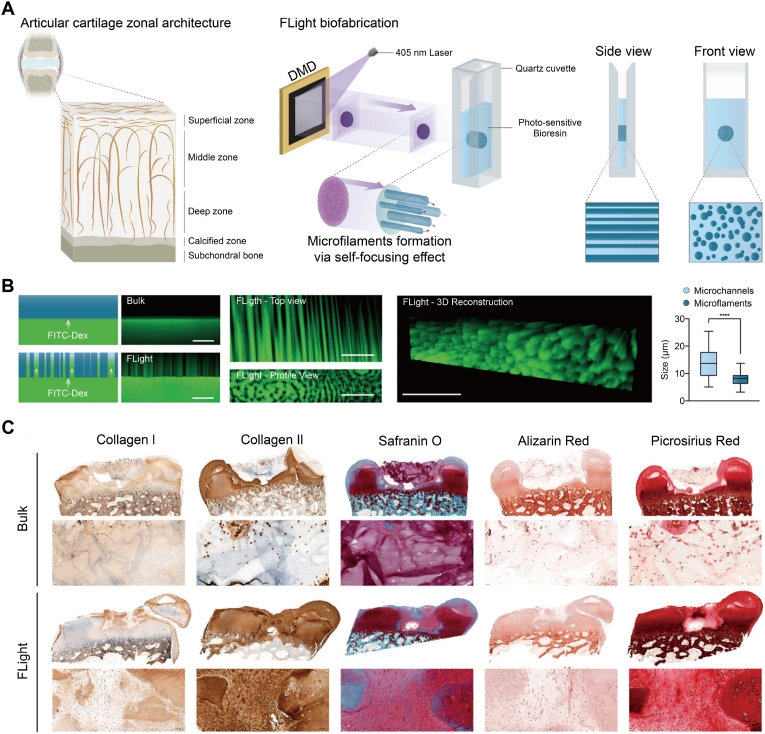
Anisotropic topography and channelized microarchitecture guided cartilage maturation. (A) Articular cartilage zonal architecture and FLight setup, where digital micromirror light projections into a photo-sensitive bioresin generated aligned microfilaments and interconnecting microchannels by self-focusing. (B) FITC-dextran infiltration showed open microchannels in FLight gels, while bulk gels remained homogeneous, as shown in top and profile views and a 3D reconstruction, and in box plots comparing microchannel and microfilament diameters. (C) Bulk *versus* FLight constructs after culture, with FLight showing stronger Col II and GAG staining, reduced Col I, minimal calcification, and aligned Col by picrosirius red. Scale bars, 100 μm. All statistical data are represented as mean ± SD (*n* = 3; ∗∗∗∗*P* < 0.0001). Reproduced with permission [132]. Copyright 2024, John Wiley & Sons.

At the scaffold level, these principles have been translated into osteochondral constructs with zone-specific architecture. Surface-topographical scaffolds tailored for articular cartilage and the underlying subchondral bone have been proposed to improve the zonal complexity of osteochondral defects [131]. As a representative example, Puiggalí-Jou et al. used a projection-based "FLight" approach to generate anisotropic, macroporous HA-NB/PEG_2_SH hydrogels within seconds [132]. Self-focusing light produced aligned microfilaments and interconnected microchannels that supported rapid dextran infiltration and directional cell guidance (Fig. 5A and B). *In situ* FLight within osteochondral plug defects yielded vertically aligned, Col II-rich neocartilage without mineralization, highlighting translational potential (Fig. 5C). Along similar lines, bilayer or multiphasic hydrogels with aligned microchannels and a mechanical gradient have been reported to support directional MSC migration and to enhance coupled cartilage−bone regeneration in osteochondral defect models, by combining topographical guidance with depth-dependent stiffness and composition [133].

In summary, surface topography acts as a geometric regulator alongside ligand presentation to control adhesion architecture, transmit forces to the nucleus, and bias MSCs toward osteogenic or chondrogenic outcomes across the osteochondral unit. Effective design matches feature size and orientation to the zone-specific demands of cartilage and subchondral bone. Nanoscale roughness or nanotubes on the bone side support integrin-mediated osteogenesis and vascularized bone formation, whereas microgrooved or pillar-like patterns on the cartilage side help organize superficial-zone-like cell alignment and promote hyaline-like chondrogenesis. At the construct scale, introducing aligned filaments and interconnected channels throughout the scaffold, rather than only on the external surface, supports directional cell guidance and rapid nutrient transport. To consolidate Sections 2 and 3, Table 1 outlines how the main biophysical cues are targeted on the cartilage and bone sides of osteochondral unit, together with their principal trade-offs for stem cell-guided regeneration.

**Table 1:** Intrinsic and interfacial design cues for stem cell-guided osteochondral regeneration.

Design cue	Cartilage side-target	Bone side-target	Comparative comment	Reference
Stiffness	A soft to moderately stiff matrix supports chondrogenesis, maintains low friction, and tolerates repetitive joint motion. Typical compressive modulus in the sub-kilopascal to tens-of-kilopascal range.	Higher stiffness to support load, resist collapse, and transmit strain to the subchondral bone Target modulus in the tens to hundreds of megapascals, close to trabecular bone	Soft substrates favor chondrogenesis, while stiffer substrates induce bone differentiation. Continuous or stepped gradients mirror the native transition in modulus.	[[22], [23], [24],^33^,^43^]
Viscoelasticity	Fast-relaxing, highly hydrated matrices dissipate impact, support rounded or columnar chondrocyte morphology, and protect the superficial zone from stress peaks.	Slower-relaxing, more elastic matrices preserve load-bearing capacity and transmit physiologic strain to osteoblasts and osteocytes.	High dissipation at the cartilage side improves comfort and chondroprotection yet may blunt mechano-sensing if relaxation is excessive. Highly elastic bone-side regions favor mechanotransduction but may keep stress away from host bone if the mismatch persists.	[[51], [52], [53],^55^,^56^,^60^]
Porous architecture	Finer pores and higher water content stabilize the repair clot, allow diffusion, and limit deep vascular invasion into the superficial cartilage zone.	Larger, open, interconnected pores favor vascularized bone ingrowth and marrow access. Pore diameters are typically in the 200−500 μm range for trabecular-like bone regeneration.	Small pores at the cartilage side help maintain a continuous, low-permeability surface but slow nutrient transport and cell migration. Large pores at the bone side accelerate bone ingrowth but reduce early mechanical strength and may require initial protection.	[68,72,73,75].
Degradation	Slow, surface-controlled degradation maintains joint congruency and lubrication while neocartilage matures. Degradation products remain near-neutral and do not trigger inflammation.	Faster, yet controlled, resorption that allows timely load transfer and bone remodeling without excessive voids or loss of support. Ionic products ideally support osteogenesis and angiogenesis.	Accelerated resorption shortens the period of mechanical support and risks subsidence or cyst formation if tissue formation lags. Very slow resorption preserves support but may lead to stress shielding and the accumulation of acidic products in polyester-rich constructs.	[[89], [90], [91], [92],^99^]
Ligand presentation	Cartilage-appropriate ligands presentation at sufficient density supports chondrogenic phenotypes and matrix retention while limiting fibroblast overgrowth. Examples include Col-derived motifs and tuned RGD densities.	Osteoconductive ligands presentation, such as Col I motifs, RGD, and bone-mimetic peptides, supports osteoblast adhesion, osteoclast activity, and strong bonding to host bone.	High ligand density improves integration and matrix production but may favor fibrocartilage or fibrous tissue if cues are not zonally tuned. Low ligand density preserves a lubricious surface yet increases the risk of poor integration and partial delamination.	[107,[109], [110], [111], [112]]
Surface topography	Smooth or gently patterned surfaces keep friction low and guide columnar chondrocyte alignment or superficial zone organisation.	Rough, microtextured, or porous surfaces promote mechanical interlock, guide bone ingrowth, and alignment along principal stress directions.	Highly rough cartilage surfaces increase wear and fibrillation under motion. Overly smooth bone interfaces reduce interlocking and may delay union. Depth-dependent topography helps split these conflicting requirements between cartilage and bone regions.	[[127], [128], [129], [130]]

Abbreviations: Col, collagen; ECM, extracellular matrix; RGD, arginine−glycine−aspartate.

## 4 Programmable and adaptive cues

Programmable and adaptive cues add a "software" layer that steers osteochondral repair. Light and thermal inputs, magnetic actuation, and ultrasound act as the primary control routes. Used together, these inputs deliver signals in space and time, adjust mild hyperthermia and mechano-sensitive pathways, and synchronize activation with rehabilitation. The overall goal is to protect cartilage in the early phase, resolve inflammation at the appropriate time, and later boost osteogenesis and integration at the interface.

### 4.1 Light and thermal stimuli

Light and temperature provide highly programmable, exogenous cues with spatial precision from micrometers to millimeters and temporal precision from milliseconds to minutes. They function as a controllable "software layer" superimposed on osteochondral scaffolds. Photochemical strategies, such as *in situ* photocuring, permit on-demand fixation of cell-loaded gels within irregularly shaped defects. Photo-thermal strategies convert optical energy into controlled, mild hyperthermia that tunes local immunity, matrix metabolism, and lineage programs [134]. A recent study showed that photocured, co-assembled hydrogels, when anchored within cartilage defects, drove hyaline-like repair *in vivo*, underscoring the clinical appeal of light as a minimally invasive trigger [135]. Parallel advances in scaffolds with photo-thermal response indicate that carefully dosed near-infrared (NIR) heating enhances osseous repair while providing antibacterial and anti-inflammatory functions at the osteochondral interface [136].

Mechanistically, light and heat act through several intersecting axes [[137], [138], [139]]. Modest temperature elevations that mimic loading-induced self-heating in articular cartilage, in combination with cyclic compression, enhance chondrocytes' perception of mechanical cues. *In vitro* thermomechanical regimens led to greater matrix deposition across constructs with different stiffnesses, underscoring heat as a physiological co-signal for cartilage anabolism [140]. Consistently, Nasrollahzadeh et al. showed that recreating the post-loading temperature rise of native cartilage enhanced TRPV4-dependent chondrogenesis in engineered constructs, confirming physiological thermogenesis as a meaningful co-cue for cartilage repair [141].

Photo-thermal modulation in osteochondral repair can be conceptually categorized into three representative strategies. The first entails thermally or NIR-gated dual-mode release in which an NIR-responsive, hydrogel-coated mesoporous bioactive-glass scaffold was engineered to provide a baseline release of a parathyroid hormone derivative. Additional NIR irradiation triggered supplemental release, which enhanced angiogenesis and bone regeneration. This outcome supports photo-thermal gating as a workable strategy for bone support in osteochondral settings [142]. The second strategy involves regulating the microenvironments through localized photothermal heating. Black bioceramic scaffolds with micro- and nanostructured surfaces generated a localized, mildly hyperthermic microenvironment under NIR. This condition accelerated repair of osteochondral defects *in vivo* and supported the use of controllable photo-thermal warming to create a more pro-regenerative interface [143]. The third strategy focuses on photo-thermal regulation of redox balance and ECM homeostasis. A photo-thermal hydrogel incorporating Mn_3_O_4_ nanoparticle scavenged excessive reactive oxygen species (ROS) and normalized ECM metabolism in a degenerative disc model under NIR stimulation. These findings illustrate that light-responsive redox modulation restores matrix homeostasis in cartilage-like tissues. The same principle is conceptually applicable to osteochondral repair, where oxidative stress and catabolic signaling impede MSC-derived chondrogenesis [144].

Translational considerations in light/thermal-responsive osteochondral scaffolds emphasize controllable deployment and compatibility with minimally invasive or arthroscopic delivery. A cell-free bioactive hydrogel with cross-link of multiple hydrogen bond displayed rapid shape memory at body temperature, allowing the injected material to recover its preset shape *in situ* and conform to cartilage defects, thereby improving retention under joint-like loading and simplifying intra-articular placement [145]. For photothermal actuation, NIR systems need controllable heating and work best with minimally invasive activation. Wu et al. designed a GelMA/methacrylated alginate (AlgMA) hydrogel with deferoxamine (DFO)-loaded polydopamine-coated black phosphorus (BP@PDA) nanosheet that showed stable 808-nm laser-responsive mild photothermal effects and NIR/pH dual-triggered release *in vitro* and *in vivo*. Repeated NIR exposure induced mild local hyperthermia, reduced inflammatory and oxidative stress, promoted M2 macrophage polarization and revascularization, and accelerated bone repair in rat defects. This stimulus-responsive setup shows a practical path to light- and heat-responsive scaffolds for osteochondral applications [146].

Overall, light/thermal platforms for osteochondral repair should be built around physiologic, nonablative heating. Real-time thermal monitoring helps reproduce the thermomechanical cues that enhance matrix deposition and TRPV4-dependent chondrogenesis in engineered cartilage constructs. Photo-thermal elements should absorb in NIR ranges compatible with minimally invasive delivery, and the scaffold phase should offer photo-thermal-gated release or temperature/shape-responsive fixation to fit irregular cartilage defects. Appropriately dosed NIR further supports immunomodulation, ROS reduction, and revascularization at the osteochondral interface, as shown in multifunctional hydrogels that generate a pro-regenerative microenvironment and accelerate bone repair.

### 4.2 Magnetic and ultrasound stimuli

Magnetic and ultrasound stimuli provide programmable, spatiotemporally precise control layers that overlay osteochondral scaffolds without altering baseline architecture [[147], [148], [149]]. In magnetic systems, superparamagnetic nanoparticles (MNPs) or magneto-responsive microstructures embedded in hydrogels or composites transduce alternating or rotating fields into local forces and torques. These effects enable remote guidance, assembly, and activation of stem cell niches *in situ* [150,151]. Ultrasound is a noninvasive actuator that can be overlaid on cartilage-directed hydrogels to trigger *in situ* gelation and ROS-sensitive drug release, enabling on-demand therapy in joint-like microenvironments. Low-intensity ultrasound applied to piezoelectric or magneto-responsive hydrogels also delivers mechanical and electromechanical cues that support chondrogenic differentiation even under inflammatory conditions, making ultrasound a remotely addressable option for localized, repeatable activation of osteochondral scaffolds [152,153].

Magnetic actuation in joint-related regenerative systems works mainly through two routes. The first is field-guided positioning and assembly. Magnetically responsive Janus hydrogel microrobots are steered by an external magnetic field to the tendon−bone interface and maintained in a defined orientation, enabling local delivery of bioactive ions and *in situ* reconstruction of structural and phenotypic gradients. The second is a magneto−mechanical transformation. Cartilage-on-a-chip platforms with magneto-responsive layers turn external magnetic fields into local deformations that are transmitted to chondrocytes, leading to directed changes in inflammation- and matrix-related gene expression and demonstrating that magnetic loading is effectively sensed by cartilage cells [154,155]. Ultrasound provides a noninvasive energy source for activating engineered scaffolds at bone defect sites. In ultrasound-triggered functional systems, exposure to a defined ultrasound regimen initiates cascade responses and controlled release, restoring MSC migration, survival, and osteogenic differentiation across different healing phases, thereby accelerating bone regeneration without global heating [156]. Ultrasound also provides a remote trigger to permeabilize or disrupt engineered hydrogel microspheres, releasing payloads, such as oxygen (O_2_) or osteoinductive magnesium ion (Mg^2+^), directly into the defect zone. Repeated insonation enables staged, spatially addressable dosing that supports angiogenesis and bone regeneration throughout repair [157,158].

**Fig. 6:**
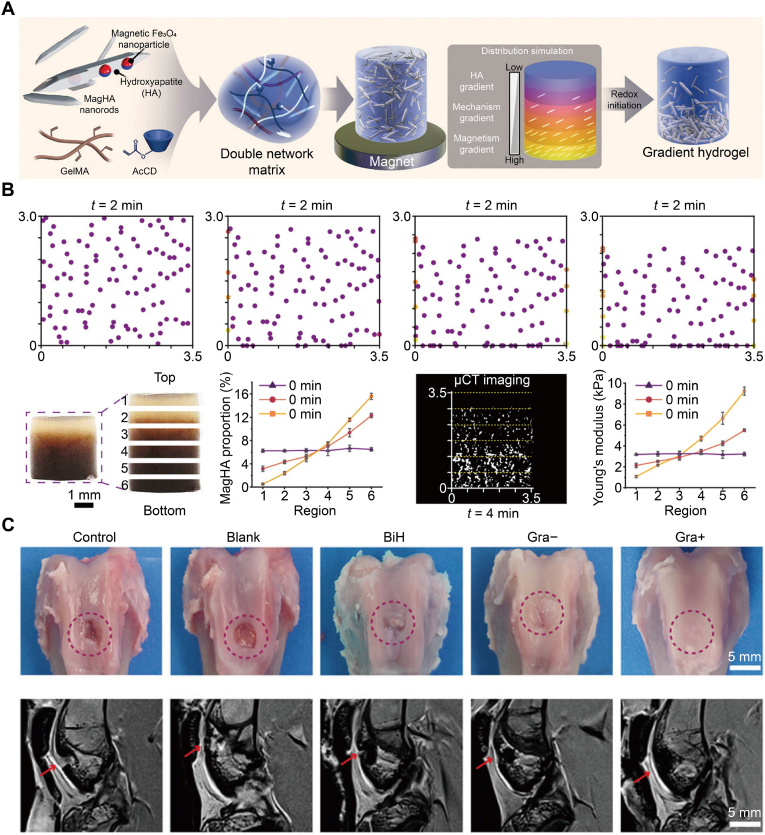
Magnetic field guidance established multilevel mineral and stiffness gradients that drove osteochondral repair. (A) Double-network GelMA/AcCD matrix containing superparamagnetic hydroxyapatite nanorod was magnet-driven and redox-cross-linked to form continuous gradients in mineral content, stiffness, and magnetism. (B) Simulation and experiments showed time-dependent bottom-enriched MagHA distribution with visible color gradient, Micro-CT validation, and increasing MagHA proportion from top to bottom. (C) Rabbit trochlear defects treated with Control, Blank, BiH, Gra−, and Gra+ showed the most complete repair in Gra+ by gross view and MRI. Scale bars 5 mm. Reproduced with permission [159]. Copyright 2024, John Wiley & Sons.

As an illustrative example of magnetically programmed gradients translating to osteochondral repair, Zhang et al. created a multilevel hierarchical hydrogel in which brief magnetic exposure patterned superparamagnetic hydroxyapatite nanorods into a continuous mineral-mechanical-magnetic gradient that matches the cartilage-to-bone profile (Fig. 6A) [159]. Time-lapse simulations and experiments showed rapid redistribution (∼4 min), leaving a ∼0.5 mm cartilage-facing layer depleted of magnetic hydroxyapatite (MagHA) and progressively higher mineral content toward the base, yielding depth-dependent stiffness (Fig. 6B). In a rabbit full-thickness osteochondral defect, the gradient construct combined with a modest static field produced smooth, hyaline-like cartilage with bone-plate closure and superior macroscopic and MRI outcomes *versus* blank, biphasic, or no-field controls (Fig. 6C).

Structurally, four-dimensional (4D)-printed magneto-responsive hydrogels are designed to reconfigure under external magnetic fields, conforming to auricular cartilage defects and maintaining mechanical properties that support cartilage formation, thereby improving fit and retention during regeneration. The magnetically controllable, multifunctional construct also provides antibacterial and anti-inflammatory benefits while supporting cartilage formation, indicating a field-triggered route to shape-adaptive cartilage scaffolds [160]. For ultrasound, Xu et al. encapsulated O_2_-enriched, selenium-containing nanoparticle in a GelMA matrix, and ultrasound irradiation enlarged the pore network, triggering rapid selenium release, enhancing intracellular selenoprotein expression, activating Wnt/β-catenin signaling, and ultimately promoting osteogenic differentiation of BM-MSCs. This example illustrates that acoustically modulatable, in-defect hydrogels may be externally scheduled to deliver bioactive components that stabilize stem cell homeostasis and accelerate repair of bone defects [161].

Translational pathways leverage existing hardware and clinical experience. Low-intensity pulsed ultrasound (LIPUS) is already in clinical use. Preclinical studies have shown that properly parameterized LIPUS inhibits YAP/RIPK1/NF-κB signaling and restores impaired autophagy, which reduces inflammation and cartilage degeneration in post-traumatic knee osteoarthritis models. These results highlight ultrasound as a feasible external trigger for intra-articular therapies [162]. On the magnetic side, osteoarthritis hydrogels integrated with superparamagnetic iron oxide nanoparticles (SPIONs) have been developed as MRI−visible theranostic scaffolds, allowing real-time, noninvasive monitoring of tissue regeneration. Magneto-mechanical studies in osteoarthritis also showed that externally applied, time-varying magnetic fields remotely drive stem cell aggregation and chondrogenic signaling in the joint without implanted leads. These preclinical data suggest that wireless magnetic targeting of intra-articular biomaterials is feasible, although field parameters still need to fit clinical safety windows [150,163]. Importantly, these programmable stimuli align with mechanical loading and perfusion (Sections 5.1 and 5.2). Cyclic compression amplifies streaming potentials and local strain, whereas perfusion improves acoustic coupling and heat dissipation, enabling lower input doses and broader safety margins.

In summary, constructs that respond to light, thermal, magnetic, or ultrasound inputs provide an additional, programmable, and noninvasive control layer on top of osteochondral scaffold architectures. Photochemical and photo-thermal inputs first help stabilize or locally heat soft, hydrated regions close to the cartilage surface. In this early period, doses remain low, and sessions remain short, so that mild hyperthermia or photoactivation supports chondrogenesis and dampens inflammation without damaging a still-fragile construct. As subchondral regions become stiffer and more vascularized, magneto-responsive and ultrasound-responsive elements in deeper, more mineralized zones take on a greater role. Field strength and duty cycle then shift toward patterns that drive drug release, matrix remodeling, or micro-scale deformation, mainly in these load-bearing regions.

In this view, timing is part of the material design. Soft, fast-relaxing hydrogels and lubricated cartilage-side coatings pair naturally with early photo-thermal or low-intensity ultrasound regimens. Stiffer, porous, bone-facing frameworks pair with later, more focused magnetic or acoustic actuation. Therefore, external dynamic cues work best when they align with the phases of tissue healing and rehabilitation and when their spatial cues match the gradients in stiffness, porosity, and degradation that are already encoded in the scaffold.

## 5 Exogenous dynamic cues

Exogenous dynamic cues add time-structured forces, flows, and fields that guide osteochondral repair. Dynamic mechanical loading, hydrodynamic shear, perfusion, and electrical stimulation act as the primary design axes. Together, they reproduce waveforms at the scale of gait, drive Ca-linked mechanotransduction and YAP/TAZ activity, improve convective transport, and generate motion-coupled bioelectric signals.

### 5.1 Dynamic mechanical loading

Dynamic mechanical loading (DML), encompassing direct compression, hydrostatic pressure, shear, and tensile regimens, is identified as a principal mechanical stimulation approach to enhance tissue-engineered articular cartilage constructs [164]. When applied within narrow, well-defined ranges of magnitude and duration, DML improves mechanical, structural, and cellular properties, yielding anisotropic, mechanically robust tissues that more closely resemble native articular cartilage [165].

At the mechanistic level, cells interpret dynamic mechanical loading primarily through mechano-sensitive ion channels, in addition to the canonical adhesion- and cilium-based pathways. TRPV4 transduces physiologic dynamic compression into Ca^2+^ oscillations that drive anabolic and metabolic programs in chondrocytes. Piezo1, by contrast, functions as a load-responsive regulator of chondrocyte fate during endochondral ossification and osteophyte formation, and its expression and sensitivity are further heightened by osteoarthritis-relevant inflammatory signaling. Such inflammatory co-signals lower the activation threshold of Piezo1 and create a feed-forward susceptibility to pathological amplification under excessive or abnormal strain [[166], [167], [168]]. Downstream, the Hippo-YAP/TAZ axis functions as a mechano- and inflammation-sensitive hub in joint tissues. Mechanical cues that remodel the actin cytoskeleton and activate RhoA/ROCK feed into the core Hippo kinases to control YAP/TAZ nuclear localization, and YAP/TAZ in turn interact with MAPK/ERK and other joint-relevant pathways to regulate chondrocyte proliferation, matrix anabolism and catabolism, and lineage decisions along the osteochondral spectrum [169,170].

Most of these mechanistic insights have been obtained from articular chondrocytes or BM-MSCs. However, ADSCs are increasingly used as an accessible cell source for cartilage repair, and their responses to dynamic biophysical cues are beginning to be clarified. Dynamic compression or cyclic hydrostatic pressure within physiological ranges enhances ADSC chondrogenesis in 3D scaffolds, upregulating the expression of *SRY-box transcription factor 9 (Sox9)*, *Col II*, and *ACAN*, and proteoglycan deposition, whereas inappropriate loading regimes (for example, excessively high magnitudes or prolonged loading) dampen chondrogenesis or bias cells toward hypertrophic or osteogenic phenotype [171,172]. Studies also implicate TRPV4- and Piezo1-mediated Ca^2+^ influx and YAP/TAZ nuclear shuttling in ADSCs mechanotransduction, indicating that ADSCs share core mechano-sensing modules with other MSC sources while retaining lineage- and context-specific responses [173,174].

**Fig. 7:**
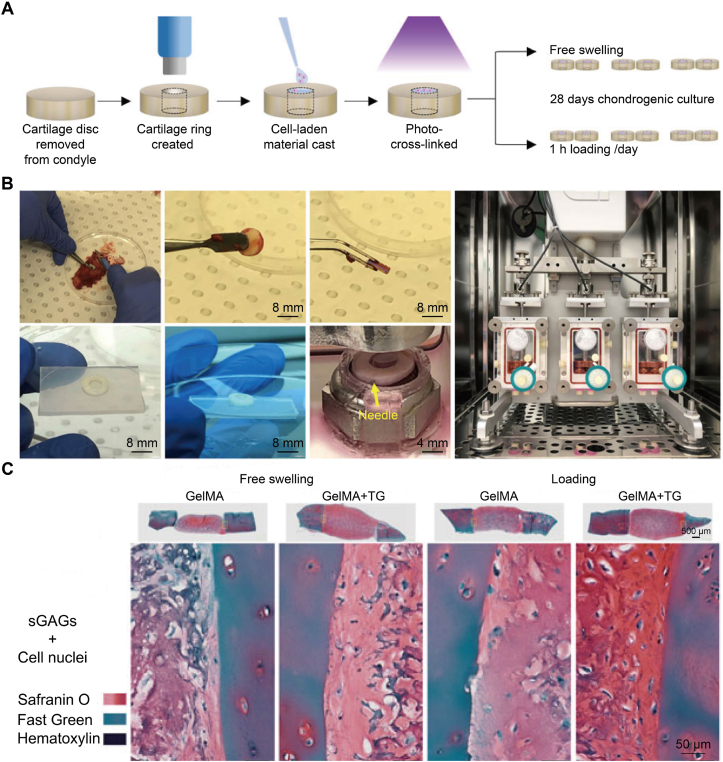
Dynamic mechanical loading enhanced matrix deposition in an *ex vivo* cartilage repair model [175]. (A) Workflow in which a cartilage disc was excised, a ring defect was created, a cell-loaded GelMA or GelMA plus transglutaminase hydrogel was cast and photo-cross-linked, then constructs were cultured for 28 days under free swelling or with daily cyclic compression. (B) Representative preparation and bioreactor setup, including cartilage ring formation, hydrogel filling, fixation with a PDMS holder and fine needle, and operation of a three-chamber compression bioreactor. (C) Safranin O/Fast Green/Hematoxylin staining after culture showed sGAG-rich matrices and cell nuclei in both free-swelling and loaded groups.

Building on these mechanistic insights, researchers have evaluated the translational value of dynamic mechanical loading (DML) in a context that reflects human osteochondral tissue. Investigators used a ring model of cartilage defects prepared *ex vivo* from human femoral condyles [175]. The defects were filled with GelMA modified with glycidyl methacrylate and carrying stem cells, either with a transglutaminase-based adhesive or without, and the constructs were cultured in a medium that promoted chondrogenesis. The constructs were assigned to one of two conditions. They were either allowed to swell freely or subjected to cyclic compression for 1 h per day for 28 days in a bioreactor designed for this purpose (Fig. 7A and B). Dynamic loading generated continuous tissue at the interface that stained strongly for Safranin O and Col II, and this tissue showed better integration than the tissue in constructs that swelled freely (Fig. 7C). The addition of transglutaminase-based adhesive provided slight additional improvement. Taken together, these data show that compression sequences defined by the loading waveform shape the interface between the hydrogel and tissue into a geometry relevant to clinical repair. For osteogenesis, Ghasemzadeh-Hasankolaei et al. showed that high cyclic hydrostatic pressure applied to MSCs cultured in liquefied microcompartments accelerated osteogenic differentiation and mineral deposition, underscoring dynamic hydrostatic loading as an effective osteoinductive cue [176].

Translational routes are converging along three lines. First, *ex vivo* mechanical preconditioning in compression, traction, or shear bioreactors before implantation remains central to functional tissue maturation and is increasingly miniaturized on joint or osteochondral chips that deliver tissue-specific strain in a controlled manner [50,177]. Second, programmable rehabilitation after cartilage repair is moving toward earlier range-of-motion goals through individualized, progressive loading. Recent expert consensus indicates that initiating joint motion as early as postoperative day 1 is safe in appropriate cases, whereas weight-bearing should be adjusted based on lesion location and concomitant procedures [178]. *In vivo* studies with biomimetic osteochondral scaffolds have also shown that partial, carefully controlled postoperative loading improves scaffold healing and integration and reduces limping, underscoring that graded loading regimens should inform clinical weight-bearing prescriptions after cartilage or osteochondral repair [179]. Third, dynamic mechanical loading acts synergistically with controlled growth factor delivery. In hydrogels containing TGF-β-loaded ALG microspheres, loading during culture enhanced cartilage-like matrix deposition over static conditions, indicating that "loading plus factor" co-application better steers chondrogenesis and limits unwanted differentiation *in vitro* [180].

In summary, DML is most effective when the loading is tuned to an appropriate level rather than pushed to the maximum. The loading should reproduce joint-like cues that chondrocytes and MSCs sense through TRPV4, Piezo1, and the Hippo-YAP/TAZ pathway. Fast-relaxing, tough hydrogels reduce peak stresses while still transmitting mechanical work. Dynamic loading is compatible with controlled delivery of bioactive factors, such as TGF-β released from microspheres, to reinforce chondrogenesis. It is also compatible with stepwise postoperative loading to promote integration. Because inflammation lowers the activation threshold of Piezo1 and increases the risk of maladaptive responses, aggressive loading should be paired with anti-inflammatory interventions or interventions targeting the relevant channels. When feasible, prescribed loading regimens should first be evaluated on human tissue-based platforms or on osteochondral-on-chip systems that impose tissue-specific strain. After these evaluations, the regimens should then be introduced into *in vivo* models.

### 5.2 Hydrodynamic shear and perfusion

Hydrodynamic shear and perfusion are dynamic biophysical inputs that turn an otherwise inert scaffold into a living reactor in which mass transport and mechanical signaling are coordinated [[181], [182], [183]]. In osteochondral constructs, perfusate moves through macro- and microchannels and the pore network to generate interstitial shear at the cell−matrix interface [184]. At the same time, it establishes O_2_ and nutrient gradients that mirror the *in vivo* contrast between a highly vascularized bone layer and the hypoxic cartilage zone. Recent musculoskeletal organ-on-chip and microfluidic scaffold platforms have shown that physiologically relevant flow and cyclic mechanical cues can be applied with high spatiotemporal precision. These platforms reproduce joint-like loading and lubrication regimes *in vitro* and use the resulting *in vivo*-style readouts to set loading parameters for stem cell-loaded osteochondral models [185].

When MSCs are exposed to fluid shear, cytoskeletal tension increases, and the integrin/FAK-RhoA/ROCK-Hippo signaling pathway is activated. This activation promotes nuclear accumulation of YAP/TAZ and induces the expression of osteogenic transcription factors, including Runx2. At the same time, fluid shear initiates Notch signaling. Activated Notch signaling further strengthens the osteogenic program, and this reinforcement depends on the magnitude and duration of flow [186,187]. Recent organ-on-chip platforms for musculoskeletal research and perfusion-based systems demonstrate that O_2_ tension, like mechanical cues, can be precisely controlled in *in vitro* musculoskeletal models. By regulating O_2_ supply across individual compartments, researchers approximate tissue-specific microenvironments and coordinate O_2_ delivery with flow-based culture to sustain readouts relevant to tissue regeneration [188].

Applying these principles, perfusion bioreactors fitted with 3D-printed scaffolds support MSC culture under sustained convective transport and mechanotransductive conditions. They also markedly increase the yield of extracellular vesicles while preserving their pro-regenerative bioactivity. These vesicles provide a paracrine resource suitable for osteo-anabolic reinforcement and as a preconditioning stage before implantation [189]. On the scaffold side, interconnected and perfusable microchannel networks (on the order of hundreds of micrometers) can be embedded in architectures formed by nanofibers or by printing. These channels accelerate vascular ingrowth and inosculation and enhance mass transport within the graft. They also support regeneration of vascularized bone, which is especially important for the highly vascular subchondral compartment in osteochondral constructs [190].

From a translational perspective, closed-loop perfusion platforms are increasingly compatible with GMP-style workflows and can be used to generate cell-loaded osteochondral grafts with real-time pressure and flow monitoring. At the same time, advances in perfusable and pro-angiogenic scaffolds, including vascular guides that present morphogens in a sustained manner under flow, provide practical strategies to achieve rapid anastomosis with host vessels on the bone side after implantation [191]. Organ-on-chip platforms and perfusion-based systems that use tightly controlled flow together with adjustable O_2_ supply provide a rapid means to define shear and O_2_ targets before researchers scale up to larger osteochondral constructs. In parallel, imposed fluid flow can be combined with other remote physical inputs. Cyclic compression introduces oscillatory interstitial flow. Magneto-mechanical actuation can be added to deliver micromechanical cues with a defined temporal pattern while nutrient perfusion is maintained. This combination has been shown to promote *in situ* assembly of MSCs and to support chondrogenesis in an osteoarthritic joint model [150].

In summary, hydrodynamic shear and perfusion function as coordinated mechanical and metabolic instructions rather than only as delivery routes. Their level, frequency, and spatial pattern must be adjusted to match the transition from avascular cartilage to vascular subchondral bone. For osteochondral constructs, perfusable channel networks should direct flow toward the bone side and maintain the cartilage side at low flow or interstitial shear. O_2_ tension should be patterned in the same cartilage−bone gradient. Shear waveforms are then chosen to drive ROCK/RhoA-YAP/TAZ and Notch signaling toward osteogenic or chondrogenic outputs, as indicated by the model. A practical sequence is as follows: (i) Use organ-on-chip platforms or other microfluidic surrogates to convert joint-like shear and O_2_ profiles into *in vitro* target values; (ii) Apply these conditions to larger scaffolds that contain embedded and interconnected channels to ensure uniform perfusion and to promote rapid inosculation.

### 5.3 Electrical stimulation

Electrical stimulation is a dynamic, field-based cue that reproduces the bioelectric signals naturally generated in osteochondral tissues during joint motion [[192], [193], [194]]. Previous studies have shown that electroactive scaffolds, particularly piezoelectric ones, translate mechanical loading into localized electrical stimuli that promote stem cell chondrogenic and osteogenic differentiation across the cartilage−bone interface [[195], [196], [197]]. It is therefore necessary to coordinate material composition, scaffold architecture, and loading patterns so that electrically induced signals integrate with other time-structured cues to support functional osteochondral regeneration [198].

At the cellular level, electrical stimulation of MSCs activates Ca^2+^-dependent chondrogenic pathways and increases the expression of Sox9, Col II, and ACAN, even in the absence of exogenous growth factors. This response indicates that electrical cues directly regulate commitment to the cartilage lineage [193]. Electrical inputs also enhance MSC adhesion, migration, proliferation, and osteogenic differentiation, supplying the cell behaviors required for electrically stimulated formation of subchondral bone [199]. Bilayer scaffolds with piezoelectric and conductive components generate opposite surface charges. These opposite charges exploit electrically responsive pathways and recruit BM-MSCs. The positively charged layer promotes chondrogenesis, whereas the negatively charged layer induces osteogenesis. Together, these effects support spatially organized osteochondral repair [200].

Harnessing these mechanisms, recent self-powered scaffolds demonstrate compelling cartilage regeneration. A moisture-tolerant, encapsulation-free triboelectric scaffold with hierarchical porous microdomains generated charge under joint-like motion and rebuilt hyaline-like cartilage in a rabbit osteochondral defect model, yielding dense Col II deposition and smooth articular surfaces without external hardware [201]. For critical-sized bone defects, an implantable, battery-free bone-defect electrical stimulation system that hybridized triboelectric and piezoelectric harvesters generated biphasic pulses driven by rehabilitation exercise. In a rat femoral critical-size defect model, this stimulation accelerated osteogenesis, enhanced angiogenesis, and achieved complete defect bridging within six weeks, with transcriptomic analysis indicating up-regulation of Ca^2+^-handling and osteogenic signaling pathways [194]. Complementing mechanically transduced systems, a self-promoted electroactive, biomimetic mineralized Col scaffold that generated weak currents through interfacial electrochemistry, activated voltage-gated Ca^2+^ channels, and triggered BMP2/Smad-dependent osteogenesis. At the same time, it exerted antibacterial effects and achieved nearly complete healing of infected bone defects in rodent, rabbit, and beagle models [202].

Translationally, the most practical route is to couple electrical stimulation with standard rehabilitation. Lightweight triboelectric or piezoelectric harvesters and electroactive scaffolds that respond to patient movement deliver on-demand pulses without batteries or percutaneous leads, improving compliance and enabling closed-loop therapy. Device-level roadmaps emphasize degradable piezoelectric polymers, such as PLLA-based copolymers, and ceramic-polymer hybrids tuned for a high *d*_33_ coefficient, fatigue resistance, and efficient interfacial charge transfer. Conductive or ionically conductive hydrogels help uniformly distribute fields within porous scaffolds [203]. Because electrical stimulation in bone and cartilage regeneration is often designed to be synchronized with mechanical inputs, it aligns naturally with dynamic-loading and perfusion regimens, as described previously in Sections 5.1 and 5.2. Mechanical deformation and fluid flow help generate or distribute bioelectric signals. As a result, the frequency and amplitude of electrical cues are set to follow a mechanical schedule that resembles rehabilitation [204].

Overall, dynamic mechanical loading, hydrodynamic perfusion, and electrical stimulation are most useful when arranged in a coordinated sequence rather than as separate add-ons. Cyclic compression and joint-like motion set the primary rhythm of forces that cells sense through TRPV4, Piezo1, and YAP/TAZ. Flow through channels and pores links this rhythm to O_2_ and nutrient delivery and to waste removal. Electrical signals from piezoelectric, triboelectric, or conductive components sit atop these patterns and convert each step, squat, or rehabilitation exercise into short voltage pulses that bias chondrogenic and osteogenic pathways in regions the scaffold is designed to polarize toward cartilage or bone.

The timing of these inputs tracks evolution of the construct. Early after implantation, low-amplitude loading, gentle perfusion, and modest bioelectric activity support cell survival and matrix deposition inside soft, fast-relaxing cartilage-side domains. As the subchondral compartment stiffens, integrates, and begins to share load, higher strain ranges, stronger perfusion, and more frequent or localized electrical pulses help consolidate bone formation and interface strength. Viscoelastic hydrogel layers, perfusable architectures, and electroactive components are therefore chosen and arranged so that mechanical, fluid, and electrical cues amplify the scaffold's mechanical and structural gradients and follow the same rehabilitation schedule, rather than competing with them. To place these dynamic strategies side by side, Table 2 compares the main cue modalities, the types of scaffold platforms that support them, their mechanobiological targets, advantages, and current level of clinical translation.

**Table 2:** Programmable stimuli and exogenous dynamic cues for stem cell-guided osteochondral regeneration.

Dynamic cue modality	Typical scaffold platform	Main mechanobiological target	Advantage	Limitations and current translation level	Reference
Photo-thermal or light-based cues	Near-infrared responsive hydrogels, surface coatings, or embedded nanoparticles that convert light to heat or trigger photochemical reactions	Local temperature elevation, phase transitions, photo-controlled release, and activation of heat-sensitive pathways	High spatial precision and on-off controllability are suitable for focal defects accessible by arthroscopy or minimally invasive light delivery.	Light penetration is limited in large joints and deep bone. Overheating or off-target exposure remains a concern. Most data are preclinical, and osteochondral-specific clinical applications are rare.	[134,142,144]
Magnetic actuation	Hydrogels or composites loaded with magnetic nanoparticles or fibres, actuated by external static or oscillating magnetic fields	Remote control of local strain fields, scaffold deformation, and on-demand release of growth factors or ions	Contact-free, spatiotemporally controllable stimulation through soft tissues Compatibility with smart hydrogels and graded composites	Requirement for magnetic components and precise field control. MRI compatibility and long-term safety must be demonstrated. Current evidence is mainly preclinical, with a few exploratory clinical uses.	[[147], [148], [149],^159^]
Low-intensity ultrasound	Hydrogels or porous scaffolds in the joint cavity are exposed to pulsed ultrasound *via* external applicators through the skin.	Integrin-FAK signaling, Ca^2+^ influx, chondrogenic and osteogenic gene expression, and local microcirculation	Non-invasive, repeatable, and relatively inexpensive deep tissue penetration and targeting to specific joint regions Clinical data exist for fracture healing and early cartilage repair	Treatment parameters for osteochondral scaffolds are not standardised. Translation is at early clinical stages with limited scaffold-specific trials.	[152,153,156,161]
Dynamic mechanical loading	Bulk osteochondral scaffolds with tuned stiffness and porosity, combined with controlled rehabilitation protocols or continuous passive motion devices	TRPV4, Piezo1, and other mechano-sensitive channels, cytoskeletal tension, matrix deposition, and alignment in cartilage and bone	Use of natural joint motion and existing physiotherapy infrastructure Strong evidence that well-timed loading improves both bone and cartilage quality	Dosing depends on patient-specific factors and joint stability. Under- or overloading reduces benefit or causes damage. Widely used clinically, but rarely optimized explicitly for scaffold mechanics.	[164,165,175,177]
Hydrodynamic shear and perfusion	Porous scaffolds with open channels, microfluidic architectures, and bioreactor-conditioned constructs implanted into load-bearing regions	Shear-responsive pathways, nutrient and O_2_ transport, synovial−subchondral cross-talk	Enhanced mass transport in thick constructs and supports zonal differentiation *in vitro* Complementarity with gentle joint motion in early phases	Shear levels are difficult to control *in vivo* and may cause abrasion if flow paths are poorly matched to joint kinematics. Evidence is strong *in vitro* and in bioreactors, but translation to standard clinical protocols remains limited.	[[181], [182], [183], [184],^189^]
Electrical and electromechanical stimulation	External electrodes are applied across the joint, or implanted piezoelectric and triboelectric elements integrated into scaffolds that harvest joint motion.	Voltage-gated channels, osteogenic and chondrogenic signalling, maintenance of bone mass, and cartilage−bone interface integrity	Long-standing evidence for bone and cartilage responsiveness to electric fields Self-powered systems based on joint motion reduce reliance on external hardware.	Device design is complex and subject to stringent regulation. Lead placement, durability, and imaging interference must be validated. Clinical data exist for bone healing and cartilage repair, but dedicated osteochondral scaffold systems are mostly at preclinical to early clinical stages.	[[192], [193], [194]]

Abbreviations: FAK, focal adhesion kinase; MRI, magnetic resonance imaging.

## 6 Synergistic biophysical signals and immune modulation

Synergistic biophysical cues together with immune modulation establish the conditions for integrated osteochondral repair. Intrinsic mechanics and transport build the baseline, whereas programmable light, thermal, magnetic, ultrasound, and electromechanical inputs update the dose across space and time. These inputs operate in concert rather than in isolation. Cells interpret stiffness, relaxation behavior, porosity, and degradation, together with exogenous fields, to determine migration, matrix assembly, and maturation. The same physical logic also governs macrophage state and vascular signaling, thereby reducing inflammation and supporting tissue formation.

### 6.1 Synergy of biophysical cues

Successful osteochondral repair requires coordinated action of multiple biophysical cues rather than reliance on a single factor. Cells simultaneously perceive stiffness, viscoelasticity, water content, pore size, and degradation behavior, and use this integrated signal set to determine spreading, migration, and extracellular matrix production. A softer, more dissipative microenvironment on the cartilage side keeps cytoskeletal tension low and supports chondrogenic programs, whereas a gradual transition to higher stiffness and more open pores deeper in the construct allows vascular and bone-related cells to enter and mature. When these properties are arranged as a smooth gradient, the interface avoids sudden jumps in load or mass transport, which makes integration easier.

Beyond these instantaneous effects on cell spreading and matrix production, the same mechanical cues also steer how the construct remodels after implantation. In the early weeks after surgery, the engineered scaffold carries most of the joint load while fragile repair tissue begins to occupy the pores. During this phase, appropriate stiffness and viscoelastic dissipation in the cartilage region limit peak contact stresses and protect the bone−cartilage interface, yet still permit small deformations that support mechano-sensitive matrix assembly. As mineralized tissue accumulates in the deeper zone and the polymeric or ceramic framework gradually loses mechanical competence, load is redistributed from the scaffold to the newly formed osteochondral unit. If this redistribution lags behind tissue formation, as with a stiff framework that persists for too long, stress shielding and a weak interface are frequent outcomes. When a compliant phase deteriorates too rapidly, collapse of the defect region and uneven loading become more likely. In contrast, scaffolds that combine graded stiffness, viscoelasticity, and porosity with an appropriate degradation profile guide load transfer along a smoother trajectory and favor organized cartilage maturation at the surface, along with coupled bone remodeling in the subchondral region. This time-evolving synergy between intrinsic mechanics and tissue growth provides the mechanical context in which architectural features, surface chemistry, and exogenous dynamic cues exert their effects.

Architectural organization and engineered surface features further reinforce this synergy. Small, less interconnected pores in the upper region retain cells locally and protect the newly deposited matrices. Larger, well-connected pores toward the area intended for bone formation enhance nutrient transport and provide sufficient volume for vascularization and bone ingrowth. At the same time, hydrophilic or bioactive surface chemistries together with gentle microtopographies improve cell attachment and orient cell morphology, enabling cells to interpret the mechanical design embedded in the scaffold. Taken together, bulk mechanical properties, controlled porosity, defined degradation profiles, and presentation of surface bioactive cues inform each cell of its spatial position within the construct and the phenotype it should adopt.

Exogenous dynamic cues sit atop this intrinsic design, allowing it to be adjusted over time. Modalities, such as ultrasound, magnetic fields, light, or mild photo-thermal inputs, trigger localized release, increase fluid transport, or deliver short mechanical stimuli without permanently altering the scaffold. In the early post-implantation phase, low-intensity or low-frequency stimulation reduces inflammatory responses and supports deposition of cartilage-like matrices in the softer region. Later, as rehabilitation begins, the same scaffold is further stimulated to promote angiogenesis and osteogenesis in the deeper, stiffer region. In this way, the static material gradient establishes the appropriate baseline, and externally applied dynamic cues allow clinicians to align the microenvironment with each stage of healing.

### 6.2 Immune modulation by biophysical cues

Innate immune cells, especially macrophages, are mechano-sensitive. Increased matrix stiffness activates a Piezo1-YAP signaling axis that drives pro-inflammatory/M1-like polarization, whereas TRPV4 activation in human macrophages suppresses IL-1β and dampens inflammatory outputs [205,206]. On this mechanistic basis, cartilage-facing regions of osteochondral constructs are best kept in a soft-to-moderate, well-hydrated, stress-dissipative range to lessen stiffness-driven Piezo1-YAP activation and to favor more resolving, chondro-supportive macrophage phenotypes, Bone-facing regions may be stiffer but should still permit relaxation to avoid load-amplified inflammation.

Surface chemistry and architecture likewise tune innate immune responses at osteochondral interfaces [207]. Zwitterionic or phosphorylcholine-like coatings reduced protein fouling and suppressed acute macrophage-mediated foreign-body reactions on neural, cochlear, and blood-contacting implants, demonstrating the broad anti-inflammatory properties of ultra-hydrophilic layers [208]. Adding zwitterionic or otherwise ultra-hydrophilic surface layers and minimizing wear debris further attenuates macrophage-mediated foreign-body responses and lowers synovitis risk [209]. Scaffolds with larger, interconnected pores or gentle curvature promoted macrophage transition from M1 to M2, increased release of angiogenic factors, and improved osseointegration, which is desirable for the vascularized bone-side compartment [210]. Micro-grooved bioceramic surfaces further biased macrophages toward pro-regenerative phenotypes even under higher local stiffness [211]. Combining hydration-rich coatings, wear profiles that minimize debris, and porosity that guides immune responses, therefore provides a multiscale route to limit synovitis and fibrous encapsulation around osteochondral constructs [212].

Recent osteochondral repair studies reinforce this view of immune-focused scaffold design. An immunomodulatory poly(amino acid) (PAA)-based PAA-RGD hydrogel with low immunogenicity induced minimal foreign-body reaction and promoted macrophage polarization toward an M2-like phenotype *in vivo*. These immune shifts were associated with improved osteochondral repair in a rabbit knee defect model when PAA-RGD hydrogels were compared with PEG-RGD or GelMA hydrogels [213]. A LiMn_2_O_4_ nanozyme-functionalized bilayer hydrogel scaffold placed an antioxidant, cartilage-facing layer above an osteogenic, bone-facing layer. This architecture reduced a ROS-rich inflammatory microenvironment and activated AMPK signaling in subchondral bone, and in a rat femoral condyle defect model, this bilayer design enhanced regeneration of both cartilage and subchondral bone relative to control hydrogels [214]. Together, these studies highlight explicit immune design targets for osteochondral scaffolds. These targets include minimizing acute foreign-body responses at the cartilage surface, biasing macrophages toward resolving M2 states, scavenging ROS, and stabilizing osteoblast and osteoclast activity in the subchondral region. Embedding such immuno-instructive strategies within phase-specific scaffold compartments aligns compartmental mechanics with a pro-regenerative immune milieu in osteochondral defects.

Taken together, these mechanistic and *in vivo* findings indicate that mechanical magnitude, surface hydration, and spatial architecture may be co-engineered to steer macrophage phenotypes in a compartment-specific manner. This compartmental view applies across the cartilage and bone sides of an osteochondral scaffold. Immune-modulatory design in this sense does not replace biochemical cues, but works with them to create a stable, low-inflammatory microenvironment that supports tissue integration and long-term function. In practice, choices about modulus, viscoelasticity, pore geometry, coating chemistry, and antioxidant components are simultaneously choices about the immune program that the scaffold presents to the joint.

## 7 Conclusion and perspectives

Scaffolds for osteochondral regeneration are more likely to succeed when stem cells experience a coordinated sequence of biophysical cues rather than a single dominant stimulus. In this review, these cues are organized into three practical tiers: Intrinsic mechanical and transport regimes, interfacial and topographic guidance, and externally programmable dynamic stimuli. Together with key mechanotransduction hubs, such as TRPV4, Piezo1, and YAP/TAZ, this framework turns stiffness, viscoelasticity, pore architecture, and dynamic loading from descriptive material properties into adjustable design variables for osteochondral scaffolds.

In practical terms, the first design task is to build a mechanically and logistically sound scaffold backbone for cartilage, interface, and bone. This backbone should reproduce depth-dependent gradients in stiffness, viscoelastic relaxation, interconnected porosity, and degradability so that each zone experiences an appropriate load-bearing and mass-transport microenvironment. On top of this intrinsic profile, interfacial design then fine-tunes how cells read and transmit forces. Ligand density and spacing, surface chemistry, and micro- or nano-scale topographies steer focal adhesion formation, cytoskeletal organization, and zonal arrangement. Exogenous cues, such as mechanical loading, perfusion, ultrasound, magnetic or photo-thermal inputs, and electromechanical stimulation, are most effective when introduced only after this baseline is in place and used as small, well-timed corrections rather than as the primary driver.

In a clinical setting, a phase-wise strategy guides the scaffold designed with rational selection and timing of biophysical cues. In the early post-operative period, scaffolds should buffer inflammation, protect the cartilage-facing surface, maintain joint congruency, and allow diffusion. At this stage, soft, hydrated, and fast-relaxing cartilage-side layers, combined with immune modulation and low-intensity stimuli, such as modest ultrasound, gentle perfusion regimes, or mild photo-thermal inputs, support pain control and early matrix deposition without overloading the defect. During the consolidation phase, as the subchondral side stiffens and vascularizes, the same constructs tolerate higher-magnitude or more focused dynamic cues, including controlled mechanical loading and electromechanical signals. Moreover, during this consolidation phase, scaffolds with designed cues are tuned to direct stem cell recruitment and lineage-specific differentiation, thereby coordinating cartilage and subchondral bone formation. In the late remodeling phase, self-powered or externally triggered electromechanical modules help maintain bone mass and interface integrity while preserving the mechanical microenvironment needed for long-term cartilage durability.

The framework also adapts to different clinical scenarios, rather than prescribing a single universal recipe. In focal post-traumatic osteochondral defects in otherwise healthy joints, the surrounding cartilage and bone are structurally intact, and inflammation is typically transient. In this context, scaffold design may prioritize rapid mechanical restoration, early but carefully graded loading, and simple, robust material combinations. Degenerative osteoarthritis or osteochondritis dissecans presents a very different landscape, with chronic synovitis, bone marrow lesions, and metabolic alterations. In these settings, scaffolds may require stronger emphasis on viscoelastic dissipation, wear resistance, and macrophage-modulating interfaces together with more conservative activation of external stimuli. Explicitly matching scaffold architecture and stimulation schedule to lesion etiology improves the likelihood of appropriate patient selection and predictable outcomes, a point that often receives less attention in purely material-focused discussions.

Existing implants and adjunctive therapies already demonstrate, in concrete terms, how specific biophysical design choices relate to distinct clinical outcome profiles. Aragonite-based, cell-free implants with a continuous, moderately stiff mineral framework and open, interconnected pores have demonstrated sustained pain and function improvements over several years in a substantial proportion of patients compared with microfracture or debridement, in line with robust subchondral support and gradual remodeling. Representative devices are summarised in Table 3, including the tri-layer Col−hydroxyapatite scaffold MaioRegen® [[215], [216], [217]], the aragonite-based implant Agili-C™ [46,218], the biphasic Col/GAG−CaP plug ChondroMimetic™ [71,219,220], the synthetic TruFit® CB plug [[221], [222], [223]], and a biphasic PLGA/β-TCP composite used with autologous cartilage [[224], [225], [226]]. Tri-layer Col-based constructs rely on softer, degradable Col-rich stacks to bridge cartilage and bone, and their mid-term outcomes have been more heterogeneous. This pattern suggests that maintaining mechanical continuity and stable integration at the bone plate during layer resorption remains challenging.

**Table 3:** Representative clinically used osteochondral scaffolds that integrate biophysical design cues.

Product	Scaffold composition and architecture	Dominant biophysical cue	Clinical evidence	Reference
MaioRegen® (Finceramica)	Tri-layer, cell-free Col I/Mg−hydroxyapatite nano-composite, mimicking cartilage, tidemark, and subchondral bone	Depth-dependent mineral and stiffness gradient, interconnected porosity for bone ingrowth	Prospective case series and multicenter studies report significant improvements in KOOS and IKDC scores up to 5−10 years, with moderate failure and reoperation rates in demanding indications.	[[215], [216], [217]]
Agili-C^TM^ (CartiHeal)	Biphasic aragonite-based scaffold with a porous aragonite bone phase and a cartilage phase partially demineralized and coated with HA	Mineral and stiffness gradients, 3D interconnected porosity with pore size around 100 μm, and mechanical mimicry of cancellous bone	Randomized controlled trials in focal and early osteoarthritic lesions show superior KOOS and pain scores compared with microfracture or debridement at 2−4 years, with sustained benefit and acceptable safety at five years.	[46,218]
ChondroMimetic™ (TiGenix/Col Solutions)	Biphasic collagen scaffold with a cartilage phase of collagen and GAGs and a bone phase containing Col plus CaP	Compositional and porosity contrast between cartilage-like and bone-like regions, a single integrated porous body	A Single-arm clinical study in 17 knees shows approximately 95% defect fill, repair tissue T2 values comparable to native cartilage, and improved KOOS and modified Cincinnati scores at a mean follow-up of 7.9 years.	[71,219,220]
TruFit® CB plug (Smith & Nephew)	Cylindrical resorbable scaffold of PLGA with CaSO_4_ and hydroxyapatite, with distinct chondral and osseous regions	Porous polymer−ceramic composite, early mechanical support with gradual load transfer during resorption	Multiple case series and systematic reviews show short-term symptom relief but frequent incomplete osseous integration and subchondral cysts, leading to variable outcomes and eventual withdrawal from routine clinical use.	[[221], [222], [223]]
Biphasic PLGA/β-TCP osteochondral composite with minced autologous cartilage (Chiang/Tseng/Kuo)	Cylindrical biphasic DL-PLGA scaffold with a lower phase impregnated with β-TCP for bone and an upper chamber filled intraoperatively with double-minced autologous cartilage	Stiffness and composition contrast between the bone-phase polymer−ceramic and cartilage-filled phase, porous architecture for bone regeneration	A pilot study of 10 knees shows improvements in KOOS scores and hyaline-like cartilage regeneration with cancellous bone reconstruction at two years. A 5-year follow-up study and a recent randomized trial report durable clinical benefit and better KOOS outcomes than microfracture.	[[224], [225], [226]]

Abbreviations: CaSO_4_, calcium sulfate; CaP, calcium phosphate; Col, collagen; GAG, glycosaminoglycan; IKDC, International Knee

Documentation Committee; KOOS, Knee Injury and Osteoarthritis Outcome Score; MRI, magnetic resonance imaging; PLGA, poly(lactic-*co*-glycolic acid); β-TCP, β-tricalcium phosphate.

Hydrated hydrogel-based autologous chondrocyte implantation matrices provide a fast-relaxing, chondroprotective microenvironment that improves defect filling and integration, but they generally depend on an intact or surgically reconstructed subchondral plate to bear load. Taken together with the products listed in Table 3, these experiences show that stiffness gradients, pore architecture, viscoelasticity, and degradation behavior already shape how scaffolds perform in patients. How these levers are tuned is likely to decide whether a given design delivers only short-term defect filling or a stable, long-term restoration of osteochondral structure and function.

Translation of biophysical signal-driven scaffolds also encounters non-trivial regulatory hurdles. Many constructs with spatial gradients or active components fall under high-risk medical device or combination-product categories, which impose strict requirements on manufacturing reproducibility, mechanical benchmarking, and long-term surveillance. Gradient architectures must demonstrate stable stiffness and porosity profiles across batches and over clinically relevant time spans. Devices that incorporate photo-thermal, magnetic, or electrical modules need clearly defined safe exposure limits, compatibility with implants and imaging, and explicit mitigation of unintended heating or electromagnetic interference. These demands favor designs in which guidance resides in measurable mechanical and architectural features. They also argue for standardized reporting of mechanical properties, relaxation spectra, and degradation kinetics alongside clinical endpoints, so that regulators and clinicians evaluate not only "whether it works", but also how it works from a biophysical perspective.

Long-term outcomes and potential complications of such biophysical designs remain incompletely understood and deserve explicit attention rather than brief mention. If subchondral support resorbs too quickly, late subsidence or cyst formation may occur. If gradients are mis-specified, either stress shielding or local overload may drive interface failure or cartilage breakdown. Repeated photo-thermal or electromagnetic stimulation in compromised joints also raises questions that current short-to mid-term trials are not powered to answer. Systematic registries and randomized studies with follow-up beyond five to ten years, therefore, play a critical role, together with harmonized definitions of structural and clinical failure. In parallel, mechanobiology-informed *in vitro* models and animal studies should anticipate and de-risk these long-term behaviors before broad clinical adoption, especially for implants that introduce strong or repeated external stimuli.

Looking forward, design and evaluation pipelines that link mechanogenomics, human-relevant osteochondral organ-on-chip models, large-animal studies, and clinical data in a single feedback loop are likely to become increasingly important. These integrated datasets are well-suited to data-driven and machine-learning approaches that relate material composition, architecture, processing conditions, and stimulation protocols to biological and mechanical performance, as suggested in recent materials-plus-machine-learning studies [227,228]. Such pipelines help address not only what stiffness, porosity, or dynamic input is appropriate, but also when and for whom specific cue combinations are suitable in a realistic clinical setting. Ultimately, the goal is for osteochondral scaffolds to act in phases. First, spatial mechanics and transport are fixed. Second, interfacial cues instruct cells which zone they occupy. Finally, minimally invasive, rehabilitation-synchronized stimulation delivers small, timed corrections. In this view, biophysical cues become parameters that are set, measured, regulated, and tracked in individual patients over time rather than static descriptors of a material. This shift, from materials described by their properties to therapies defined by programmable biophysical signals, represents a key opportunity for the next generation of osteochondral repair strategies.

## CRediT authorship contribution statement

**Yu Gao:** Writing – review & editing, Writing – original draft. **Yaling Zhuang:** Writing – review & editing. **Tongtong Zhu:** Writing – review & editing. **Hanyang Zhang:** Writing – review & editing, Conceptualization. **Yinan Wang:** Writing – review & editing, Supervision, Funding acquisition, Conceptualization. **Fei Chang:** Writing – review & editing, Supervision, Funding acquisition, Conceptualization. **Jianxun Ding:** Writing – review & editing, Supervision, Project administration, Funding acquisition, Conceptualization.

## Ethics approval and consent to participate

None.

## Declaration of competing interest

The authors declare that they have no known competing financial interests or personal relationships that could have appeared to influence the work reported in this paper.
